# EGF receptor (EGFR) inhibition promotes a slow-twitch oxidative, over a fast-twitch, muscle phenotype

**DOI:** 10.1038/s41598-019-45567-4

**Published:** 2019-06-25

**Authors:** Margherita Ciano, Giada Mantellato, Martin Connolly, Mark Paul-Clark, Saffron Willis-Owen, Miriam F. Moffatt, William O. C. M. Cookson, Jane A. Mitchell, Michael I. Polkey, Simon M. Hughes, Paul R. Kemp, S. Amanda Natanek

**Affiliations:** 1 Department of Medicine, Imperial College London, London, UK; 2 National Heart and Lung Institute, Imperial College London, London, UK; 3 Royal Brompton and Harefield NHS Foundation Trust, London, UK; 4 Randall Division of Cell & Molecular Biophysics, King’s College London, London, UK

**Keywords:** Physiology, Diseases

## Abstract

A low quadriceps slow-twitch (ST), oxidative (relative to fast-twitch) fiber proportion is prevalent in chronic diseases such Chronic Obstructive Pulmonary Disease (COPD) and is associated with exercise limitation and poor outcomes. Benefits of an increased ST fiber proportion are demonstrated in genetically modified animals. Pathway analysis of published data of differentially expressed genes in mouse ST and FT fibers, mining of our microarray data and a qPCR analysis of quadriceps specimens from COPD patients and controls were performed. ST markers were quantified in C2C12 myotubes with EGF-neutralizing antibody, EGFR inhibitor or an EGFR-silencing RNA added. A zebrafish *egfra* mutant was generated by genome editing and ST fibers counted. EGF signaling was (negatively) associated with the ST muscle phenotype in mice and humans, and muscle *EGF* transcript levels were raised in COPD. In C2C12 myotubes, EGFR inhibition/silencing increased ST, including mitochondrial, markers. In zebrafish, *egfra* depletion increased ST fibers and mitochondrial content. EGF is negatively associated with ST muscle phenotype in mice, healthy humans and COPD patients. EGFR blockade promotes the ST phenotype in myotubes and zebrafish embryos. EGF signaling suppresses the ST phenotype, therefore EGFR inhibitors may be potential treatments for COPD-related muscle ST fiber loss.

## Introduction

Conditions such as Chronic Obstructive Pulmonary Disease (COPD), cardiac failure, type 2 diabetes, obesity and muscle wasting are major healthcare issues in ageing populations^1^. In these conditions, exercise capacity, whole-body metabolism and glucose homeostasis are impaired and since they are highly dependent on skeletal muscle, the proportion of slow-twitch (ST) to fast-twitch (FT) fibers in the large locomotor muscles has significant clinical impact^2^. This is because, in man, ST, type I, fibers are energy-efficient, mitochondria-rich, adapted for fat and glucose oxidation, and more insulin-sensitive^3^, resistant to fatigue^4^ and chronic disease-related wasting^5,6^ than FT oxidative/glycolytic IIa and glycolytic IIx fibers. A reduced quadriceps ST fiber proportion is prevalent in COPD (affecting more than half of patients with moderate to very severe lung disease)^5,7,8^, as well as in cardiac failure^6^, type 2 diabetes and obesity^9,10^. Quadriceps ST fiber proportion is an independent predictor of reduced maximal exercise capacity^5^ and also mortality^11^ in COPD. It is also an independent predictor of reduced maximal exercise capacity in patients with cardiac failure and type 2 diabetes^12,13^ and of insulin resistance^9^ and adiposity^10^. In COPD, the increased population of hybrid ST/IIa fibers in the quadriceps muscle indicate that ST fibers are switching to FT^8^. An opposite FT to ST switch can be induced in animal models with manipulation of neuromuscular stimulation patterns and forced transcription factor and co-activator expression^14,15^ but not with exercise in patients^16^. Drugs targeting relevant pathways to increase ST fibers in skeletal muscle would therefore be novel therapies for exercise limitation in COPD and metabolic disease^2,17^, and would be anticipated to reduce these patients’ susceptibility to muscle atrophy because of ST fibers relative resistance to atrophy.

Here we report that inhibition of an EGFR-driven pathway promotes the ST muscle phenotype *in vitro* and *in vivo*, and demonstrate that this pathway appears relevant to COPD-related ST fiber loss in the locomotor muscles.

## Results

### Muscle EGF transcripts are negatively associated with a ST phenotype in mice

**Figure 1:**
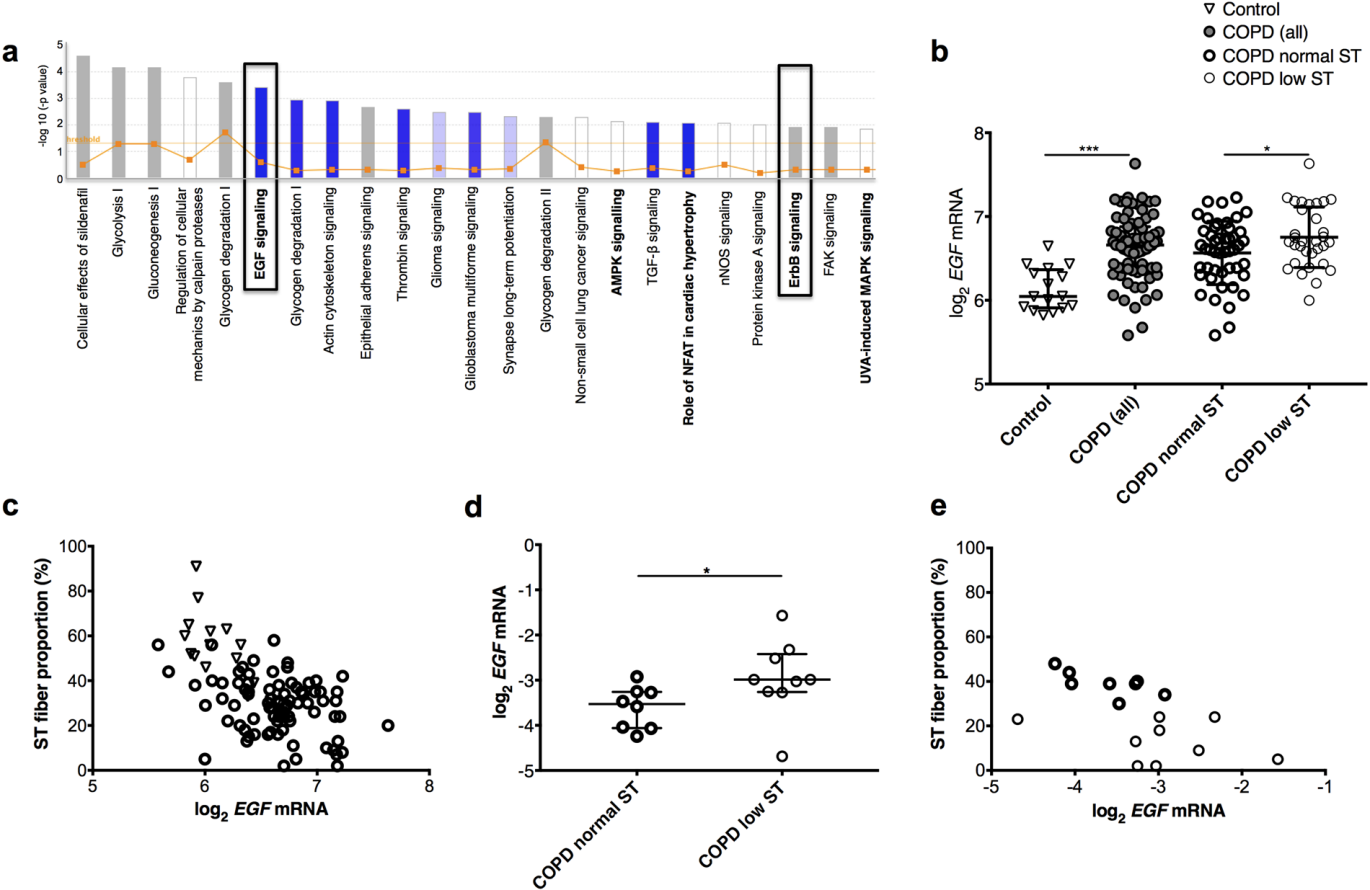
Pathway analysis of mouse data and human muscle analysis. (**a**) Pathways overlapping with genes downregulated in mouse ST versus FT IIb fibers identified through the use of IPA (QIAGEN Inc., https://www.qiagenbioinformatics.com/products/ingenuitypathway-analysis)^48^. Z-scores <0 (blue bars) and = 0 (white bars) signify more, or equal, pathway activations compared to inhibitions respectively reported in association with downregulated genes, grey indicating absence of directionality data. See methods for ratio; threshold indicates statistical significance. (**b**,**c**) Human muscle microarray data (participant characteristics in Table 1). (**b**) Quadriceps *EGF* transcripts in controls, COPD low and normal ST: patients with a ST fiber proportion <27% and ≥27% respectively. Mean (SD) and t-test results shown. (**c**) Quadriceps EGF transcripts versus ST fiber proportions (r = −0.69, p = 0.003 in controls, r = −0.56 in patients and −0.62 in patients with controls, both p <z0.0001). (**d**,**e**) QPCR data for small COPD cohort (Table 1). (**d**) Quadriceps EGF transcripts in low and normal ST groups. Median (IQR) and Mann-Whitney U-test results shown. E: Quadriceps EGF transcripts versus ST fiber proportions (r = −0.56, p = 0.019). *as for Table 1.

Pathway analysis of differentially expressed genes in isolated mouse ST and FT IIb fibers data-mined from^18^ identified EGF signaling as the top signaling pathway negatively associated with ST phenotype, together with EGF receptor (ErbB) and mitogen-activated protein kinase/extracellular signal-related kinase (ERK) signaling, the latter a key pathway activated by EGFR in response to EGF^19^ and previously implicated in muscle fiber-type regulation^20,21^ (Fig. 1a and Supplementary Fig. E1). This led us to investigate EGF and EGFR expression in muscle from healthy humans and COPD patients.

### Muscle EGF transcript levels correlate negatively with ST fiber proportion in COPD patients and healthy controls

We have previously published a genome-wide expression analysis of quadriceps biopsies from 79 COPD patients and 16 healthy matched controls from this cohort followed by a Weighted Correlation Network Analysis (WGCNA) of the results^22^. This analysis revealed six modules of co-expressed transcripts, of which two (labelled ‘yellow’ and ‘turquoise’) showed the strongest correlation with type I fiber proportion and maximal aerobic exercise capacity. By conducting an exhaustive screening of all possible glm models predicting type I fiber proportion and maximal aerobic exercise capacity from the six previously defined co-expression modules, we show here that the yellow module yields the greatest overall support, and relative to the other modules, represents the most important term in predicting exercise capacity (Supplementary Fig. E2A and E2B).

Within the yellow module documented in^22^, *EGF* has the second highest absolute module membership (MM 0.81); a measure of eigengene-based connectivity widely applied for the identification of intramodular hub genes. Also showing high levels of module membership is *SOX6* (MM 0.77) encoding the sex determining region y-related Box 6, a transcription factor shown elsewhere to suppress ST fiber development in mice^23^ and zebrafish^24^.

To explore these findings further we extracted *EGF* transcript levels from^25^ and assessed the relationship between its abundance, disease status and ST fiber proportion. COPD patients had higher *EGF* transcript levels in the quadriceps, with the highest levels in patients with a low ST fiber proportion (both p < 0.0001, Fig. 1b, normal ST proportion >27% according to reference ranges from^7^). Quadriceps *EGF* transcript abundance and ST fiber proportion were negatively correlated in patients (r = −0.56, p < 0.0001), controls (r = −0.69, p = 0.003), and in both groups when combined (r = −0.62, p < 0.0001, Fig. 1c). *EGF* mRNA levels were also negatively correlated with maximal aerobic exercise capacity measured as peak oxygen consumption on an incremental cycle test (Supplementary Fig. E2C). Array data was replicated by qPCR using RNA from separate residual quadriceps specimens from COPD patients with ST fiber proportions that were very low (median 13%, range 2–24%, n = 8) or within normal range (median 39%, range 30–48%, n = 9); 12 patients were in the microarray study, 5 were additional (Table 1). *EGF* transcript abundance was again highest in patients with a low ST fiber proportion (p = 0.036, Fig. 1d), and negatively correlated with ST fiber proportion (r = −0.56, p = 0.019, Fig. 1e) and muscle endurance (r = −0.64, p = 0.01, Supplementary Fig. E3A) in patients. Serum EGF protein levels did not differentiate patients and controls, nor patients based on ST fiber proportions (Supplementary Fig. E3D). Quadriceps *EGFR* mRNA levels were not different between patient groups, nor correlated with ST fiber proportion or *EGF* mRNA levels (Supplementary Fig. E3B,C). Together the data suggest that muscle EGF signaling as a result of EGF derived within muscle influences ST fiber proportion in humans and that increased EGF signaling in the quadriceps muscle of COPD patients may underlie ST fiber loss. We therefore investigated effects of inhibiting skeletal muscle EGF signaling *in vitro*.

**Table 1:** COPD patient and healthy control characteristics in cohort used for microarray and PCR.

	Microarray cohort				PCR cohort	
	Patients (n = 79)	Controls (n = 16)	COPD low ST (n = 32)	COPD normal ST (n = 47)	COPD low ST (n = 9)	COPD normal ST (n = 8)
Age (years)	67 (8)	66 (7)	66 (6)	68 (9)	61 (14)	75 (11)*
Sex (% males)	65	50	62	69	66	100
FEV_1_ (% predicted)	45 (18)	109 (14)****	38 (14)	50 (20)	21 (6)	48 (50)***
TL_CO_ (% predicted)	43 (17)	90 (16)****	35 (17)	48 (16)	27 (17)	54 (18)****
Quadriceps endurance (T_80_, s)	80 (35)	118 (139)*	75 (30)	85 (34)	83 (64)	90 (35)
Peak VO_2_ (ml/kg/min)	12.3 (4.0)	23.4 (7.6)****	11.6 (4.8)	12.7 (3.4)*	8.8 (3.0)	10.7 (4.5)
% ST fibers	30 (18)	54 (22)***	18 (14)	36 (11)****	13 (20)	39 (8)***
% I/IIa fibers	3 (5)	2 (6)*	3 (6)	3 (5)	2 (9)	2 (6)
% FT IIa fibers	60 (12)	40 (13)****	69 (12)	56 (12)****	69 (11)	59 (13)*
% FT IIx fibers	4 (9)	3 (6)	8 (13)	2 (6)***	8 (13)	2 (6)

Mean (SD) or median (IQR) shown, t-test, Mann Whitney U-test, and Fisher’s exact test as appropriate, *p < 0.05, **p < 0.01, ***p < 0.005, ****p < 0.0001. FEV_1_ Forced Expiratory Volume in 1 second, TL_CO_ carbon monoxide diffusion capacity, T_80_ time for force to drop to 80% of initial, VO_2_ oxygen consumption on maximal incremental cycle ergometry.

### EGFR inhibition in differentiated muscle cells increases ST gene expression

**Figure 2:**
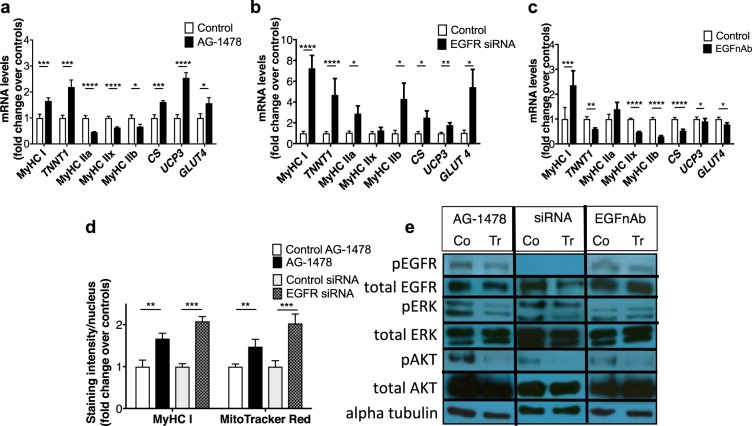
EGF pathway inhibition in myotubes. (**a**–**d**) QPCR data from mouse myotubes treated with AG-1478 (**a**), EGFR siRNA (**b**), EGF neutralizing antibody (EGFnAb) (**c**), PD98059 (**d**), or respective vehicle controls. (**e**) MitoTracker Red and MyHC I staining intensity of myotubes treated as in (**a**,**b**). Mean(SEM) shown, n = 12/group for qPCR, n = 8/group for staining (2 experiments, 2–6 different biological replicates per experiment, 2 technical replicates per sample, t-test or Mann-Whitney U-test used depending on data distribution). * as for Table 1. Representative images of Western Blot bands from samples treated with (**a**) and (**c**) or vehicle control (Co control, Tr treated) and lysed 1-hour post-single dose, and 3 days following siRNA or control vector transfection. Phosphorylated and total protein quantifications are from the same membrane.

EGF signaling blockade was investigated in C2C12 mouse myotubes using 3 approaches (i) pharmacological EGFR inhibition with AG-1478 (a selective, reversible inhibitor of the tyrosine kinase domain) (ii) *EGFR* knock-down using an siRNA and (iii) an EGF neutralizing antibody. Three days of AG-1478 increased transcripts of ST fiber-specific contractile components such as myosin heavy chain I (MyHC I, *MYH7*) and slow troponin I *(TNNT*1), and decreased FT fiber-specific transcripts, such as *MYH2*, *1* and 4 that respectively encode MyHC IIa, IIx and IIb (latter absent in man) (Fig. 2a). Increased MyHC I protein was confirmed with immunohistochemistry (Fig. 2d and Supplementary Fig. E4). AG-1478 also increased transcripts associated with mitochondrial oxidative metabolism such as citrate synthase (*CS*), mitochondrial uncoupling protein 3 (*UCP3*). Consistent with this observation, AG-1478 treatment increased MitoTracker Red staining of functional mitochondria in myotubes indicating an overall increase in mitochondrial activity (Fig. 2d and Supplementary Fig. E5). AG-1478 also increased transcripts of insulin-responsive glucose transporter 4 (*GLUT4/SLC2A4*), which is more highly expressed in ST muscle^26^ (Fig. 2a). 1 hour after AG-1478 treatment there was a very modest reduction in phosphorylated EGFR (pEGFR)/total EGFR but this was associated with reductions in downstream pERK/ERK (1 and 2) and pAkt/Akt compared to treatment with vehicle control, each time the experiment was run (n = 3, representative image in Fig. 2e and additional images and quantification in Supplementary Fig. E6A). Similar results were observed 24 hours after the final treatment (i.e. 24 hours after the third daily treatment) with AG-1478 compared to treatment with vehicle control, and also at 1 hour after treatment with AG-1478 plus 100 ng/ml EGF compared to treatment with vehicle control plus 100 ng/ml EGF (the additional EGF used to enhance the baseline signal) (Supplementary Fig. E6B,C).

Knocking down EGFR with a silencing RNA increased transcripts encoding MyHC I, *TNNT1*, and *CS*, *UCP3* and *GLUT4*, and increased MyHC I protein and staining of functional mitochondria (Figs 2b,d, Supplementary Figs E7 and E8), compared to control-transfected cells. There were additional smaller increases in transcripts encoding FT MyHC IIa and IIb (Fig. 2b) not seen with AG-1478. The siRNA knocked down EGFR protein and reduced pERK/ERK and pAkt/Akt, each time the experiment was run (n = 3, representative image in Fig. 2e and additional images and quantification in Supplementary Fig. E6A). Together with the AG-1478 data, this demonstrated that both pharmacological and genetic EGFR inhibition promoted the ST muscle phenotype *in vitro*.

Next, we reduced the concentrations of EGF in the cell culture medium (present as a result of the addition of horse serum^27^ and any secretion from cells). Addition of EGF neutralizing antibody (EGFnAb) to the medium increased transcripts encoding MyHC I but decreased all other mRNAs measured (Fig. 2c). Addition of the antibody did not reduce pERK1/ERK or pERK2/ERK2 but did cause a modest reduction in pEGFR/EGFR and pAkt/Akt 1 hour after treatment, each time the experiment was run (n = 3, representative image in Fig. 2e and additional images and quantification in Supplementary Fig. E6A). 24 hours after the final treatment (i.e. 24 hours after the third daily treatment) 24 hours after the final treatment (the third day of treatment) there was a modest reduction in pEGFR/EGFR but no reduction in markers of downstream signaling (Supplementary Fig. E6B and E6C). Therefore EGF depletion from medium decreased EGFR phosphorylation status but did not suppress downstream ERK signaling as EGFR blockade had, nor did it increase ST gene expression. This may relate to the presence of alternative EGFR ligands, which are known to have different downstream signaling patterns^28^, in the medium that maintain suppression of the ST muscle phenotype despite reduction in EGF concentrations.

#### egfra depletion increases ST fiber development and mitochondrial content in zebrafish

**Figure 3:**
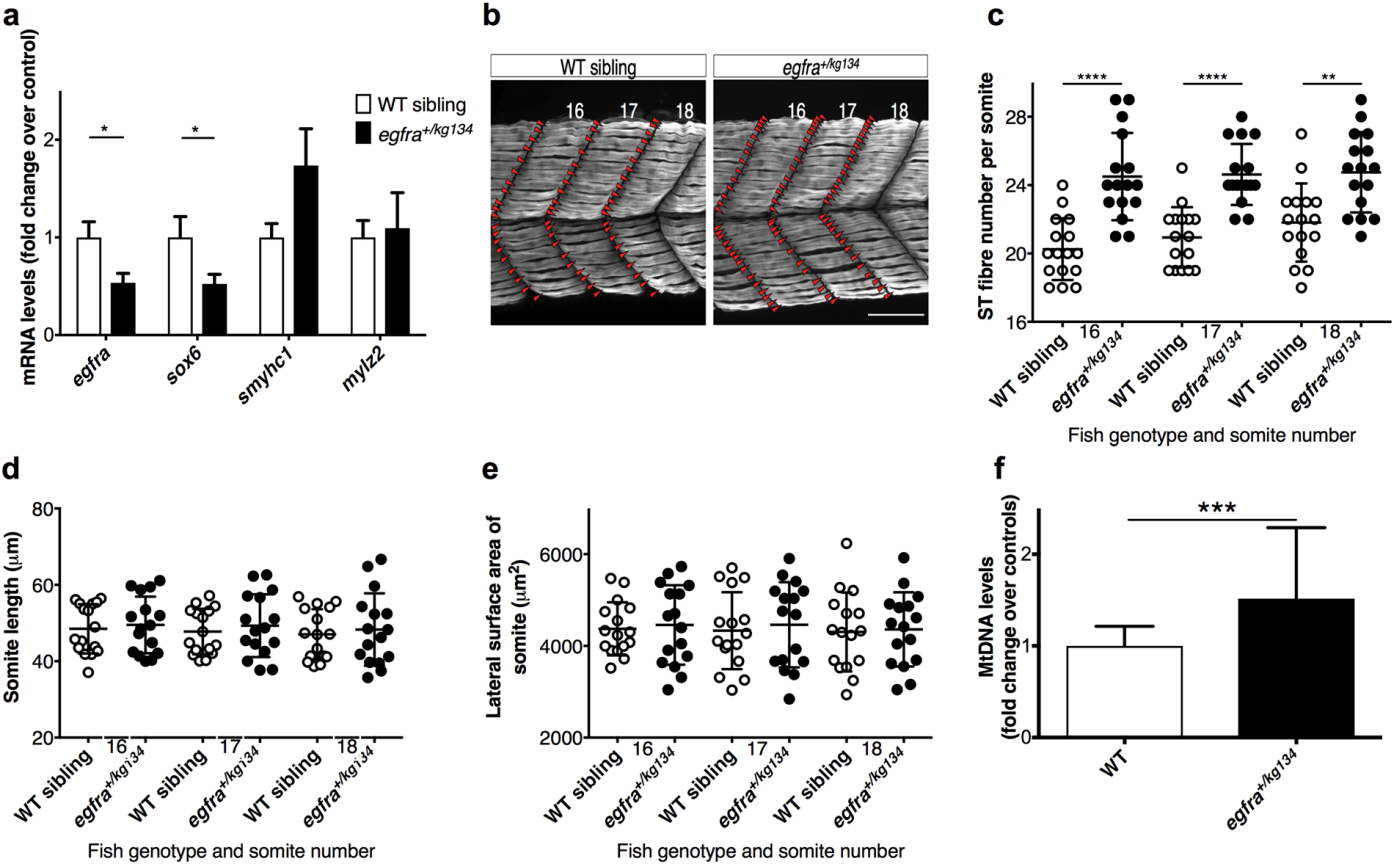
*egfra* depletion on ST fiber development and mitochondrial content. Analysis of 2 dpf *egfr*^+/*kg134*^ larvae and WT siblings. (**a**) QPCR data (n = 4 (pool of 5 larvae)/group, from 2 outcrosses)*. Smyhc1* and *mylz2* encode slow MyHC and fast myosin light chain respectively. (**b**) Maximum intensity projections of immunostained ST fibers (scale bar = 50 µm). (**c**–**e**) ST fiber number (C), length (D) and lateral surface area (E) of somites 16–18. n = 16/group (5–6 larvae, 3 crosses, Mann-Whitney U-test). (**f**) Mitochondrial DNA content n = 12/group, from 2 outcrosses. Mean (SEM) and t-test results shown. *as for Table 1.

After demonstrating that EGFR inhibition promotes the ST phenotype *in vitro*, we investigated EGFR signaling *in vivo*. We confirmed that *egfra* is widely expressed in larvae 2 dpf^29^ using mRNA *in-situ* hybridization (Supplementary Fig. E9), then generated an early nonsense mutation in *egfra* (*egfra*^*kg134*^) in zebrafish embryos with CRISPR/Cas9 (see Supplementary Figs E10 and E11 and Supplementary Information for details). Homozygous *egfra*^*kg134*^ zebrafish larvae died early, consistent with *Egfr*-knockout lethality in mice^30^. At 2 dpf, heterozygous *egfra*^+/*kg134*^ larvae expressed approximately 50% less *egfra* transcript (Fig. 3a), and had a significantly greater (16 ± 2%) number of ST fibers per somite, without somite size differing, compared to wild-type (WT) siblings (Fig. 3b–e, Supplementary Video E12). Consistent with this, transcripts of *sox6*, a suppressor of ST fiber development in zebrafish^24^ were (50%) lower (Fig. 3a), while mitochondrial DNA content was 50% greater, in heterozygotes than WT siblings (Fig. 3f). *egfra* depletion therefore promotes the ST muscle phenotype in this *in vivo* model.

## Discussion

Our results, from human muscle and varying *in vitro* and *in vivo* experiments using pharmacological and genetic approaches, suggest that increased EGF-EGFR signaling contributes to ST fiber loss in COPD patients and demonstrate that EGFR signaling blockade promotes the development and maintenance of the ST muscle phenotype. EGF, and ErbB receptor, signaling was first identified from pathway analysis of mouse data before a consistent negative association between quadriceps *EGF* transcripts and ST fiber proportions was demonstrated in healthy elderly humans and in COPD patients, and *EGF* expression was found to be higher in patients than controls, and higher in patients with an abnormally low ST fiber proportion compared to patients with a ST fiber proportion within normal range. Consistent with EGF signaling being a regulator of fiber type, EGFR blockade in differentiated mouse muscle cells in culture increased ST (including mitochondrial) gene and protein expression, effects not recapitulated by reducing EGF levels in the medium. Furthermore, in an *in vivo* model using another vertebrate species, the zebrafish, and in the context of development, we found a similar role of EGFR signaling blockade again: *egfra* depletion resulted in an increased number of ST fibers. The data suggest that the EGF-EGFR signaling mechanism is an important pathway in regulating skeletal muscle fiber type which has been conserved across species. Moreover, it raises the possibility that EGFR blockade will have some therapeutic value in COPD patients with a low quadriceps ST fiber proportion, where this is associated with exercise limitation and mortality and may also predispose patients to muscle wasting as FT fibers are more prone to COPD-related atrophy than ST fibers^5,31^. EGFR inhibitors, such as gefitinib and erlotinib, are in the clinic as cancer treatments. Since our data suggests that modest EGFR blockade modifies muscle phenotype, lower, and hence more tolerable, doses than those used in cancer treatment regimens (which aim for total downstream signaling blockade^32,33^) may be efficacious in COPD patients. Further data supporting this hypothesis could be gained from investigating muscle ST fiber proportion in patients receiving EGFR inhibitor treatment for cancer.

The data is novel because no previous data implicates EGFR activation in skeletal muscle fiber-type specification nor identifies a signaling mechanism related to ST fiber loss in COPD that is targetable by clinically available pharmacotherapies. Our results are, however, in keeping with the finding that Scube2 proteins, which contain EGF-like repeats, are pivotal for ST fiber development in zebrafish^34^, and previous literature implicating ERK1/2 signaling (which is activated by EGFR signaling^19^) in fiber-type specification. Consistent with our results, Shi *et al*. have reported greater activity of ERK in mouse FT, compared to ST, muscles^35^, and that ERK inhibition increases slow myosin expression *in vitro* and in non-regenerating mouse and rat FT muscles^20^. However, ERK1/2 activation (rather than inhibition) has been implicated in re-establishing the slow muscle programming in rat soleus muscle (but not fast muscle) following denervation-induced ST to FT switch^21^. This suggests the context and presence of additional regulators dictates fiber-type effects in response to ERK signaling, and is consistent with the fact that ERK signaling is activated by a number of upstream pathways, not only EGFR signaling. For example, insulin growth factor 1 signaling activates ERK in skeletal muscle^36^ but has not previously been described to induce fiber-type specific gene expression^37^. Our data suggest a reduction in pAkt in response to EGFR inhibition, raising the possibility that treatment with an EGFR inhibitor would reduce muscle protein synthesis and thereby muscle mass. How likely such an outcome would be, in a clinical context, is open to conjecture, as is any impact on physiology. Multiple regulators signal via Akt *in vivo* each with their own subset of outcomes. Inhibiting one of these regulatory pathways (in this case the EGFR pathway) may have minimal effect on overall Akt activity but a specific impact on the outcomes related to that pathway. As IGF-1 appears to be the major regulator of Akt in muscle influencing protein synthesis, EGFR may not have a significant impact on muscle mass. Furthermore, the positive effect on oxidative phenotype may outweigh any reductions in muscle mass on clinically relevant endpoints like exercise performance on submaximal exercise tests remains to be seen.

The cell culture differences resulting from varied disruption of EGF signaling warrant discussion. AG-1478 treatment suppressed expression of FT IIb myosin transcripts unlike the EGFR siRNA, suggesting that this may have resulted from effects of AG-1478 on ErbB2-4 receptors or off-target effects. The relevance to humans, given that they do not express MyHC IIb is debatable. EGFnAb in the medium decreased expression of ST and mitochondrial markers, apart from ST myosin, in contrast to EGFR blockade. An inadequate decrease in EGF concentrations from the medium is one possible explanation of the findings. If the main EGF source for the muscle was not accessible to the EGFnAb, which could occur if cell-derived EGF was retained in the matrix, is one possible explanation. This suggestion is consistent with sequestration of muscle-derived EGF within the extracellular matrix during regeneration without circulating EGF rising^38^ and with our human data correlating muscle, but not circulating, EGF with ST fiber proportions. Alternatively, or additionally, the EGFnAb experiment results could reflect non-EGF mediated EGFR (and other ErbB receptor) activation, released by the removal of competition of these ligands with EGF. Since signaling downstream of EGFR is specific to the particular ligand^28,39^ it would not be surprising that the phenotype produced differs to that expected with reduced EGF-induced EGFR activation. In any case, this experiment suggests that the effects of EGFR blockade and systemically administered EGF neutralizing antibody on skeletal muscle phenotype differ.

### Limitations

The study focused on the role of EGF signaling in fiber-type specification, and the therapeutic potential of inhibiting it, hence the focus on loss-of-function experiments. The focus was on describing the clinical phenotype of increased ST gene expression/fibers with EGFR inhibition, rather than describing mechanistic aspects such as which of the known signaling mechanisms downstream of EGFR responsible for the phenotype change, as this is not relevant to translation to clinical studies of EGFR inhibitors. However, we appreciate that these mechanistic studies and also gain-of-function studies would be interesting and produce more indepth insights. Cell culture has limitations in modelling what happens *in vivo*, regardless of cell type. We used C2C12 myotubes rather than primary cells from humans because this was simpler, associations between EGF and fiber type were the same in mouse and human, and C2C12 myotubes beyond 6 days of differentiation express more FT than ST myosin^40^ resembling the pattern in patients. We used AG-1478 rather than an EGFR inhibitor in the clinic, because the likely required dose ranges were well-outlined and the mechanism of action of AG-1478 and the commercial inhibitors are identical^41^. We appreciate that there were differences in the experimental design of the EGFR inhibitor and siRNA cell culture experiments, necessitated by the difficulty of introducing genetic material into fully differentiated myotubes, but were reassured by the similarities between findings despite this. The zebrafish was a practical model to address our question because of ease of breeding and tracking ST fibers which lateralize within the somite early in development^42^. Had we performed the experiment in mice, this would have necessitated generating a muscle-specific inducible knockout of EGFR. This is because of the pre- and post-natal lethality of early EGFR knockout and also the ubiquitous role of EGFR in many tissues, such that that knockout in non-muscle tissues may have significant secondary effects on muscle. The use of a zebrafish rather than a mammalian model, and a developmental/myogenesis model rather than an adult *in vivo* model, could be viewed as limitations. However, the observation that inhibition of EGF signaling promotes the ST phenotype in this different context reassured us of the consistent role of EGF in ST fiber specification. It could also be argued that there is a more direct relevance of our developmental/myogenesis data. Fast-to-slow fiber type transitions in adult animals, at least when induced by changes in electrical stimulation, have been shown to involve recruitment of satellite cells, such that ablation of satellite cells by irradiation attenuates the fiber type transitions^43,44^. In quadriceps of COPD patients there is evidence of increased satellite cell recruitment to regenerating muscle fibers than in controls, in the form of increased numbers of fibers with centralised nuclei^45–47^. Therefore, if EGFR inhibition promotes commitment of satellite cells to a ST muscle phenotype this would promote FT to ST fiber-type switching in adult muscle. We didn’t replicate the work in a mammalian model as the data already gathered appears to be sufficient to proceed to the next step -a prospective observational study of muscle fiber type changes in cancer patients treated with EGFR inhibitors.

In conclusion, we describe that EGFR inhibition promotes the ST muscle phenotype, a mechanism that appears conserved across species and scenarios of both myogenesis and fiber maintenance, and that increased EGF signaling is associated with a loss of the ST phenotype in COPD patients as well as being negatively associated with the ST muscle phenotype in mice. These findings suggest that studies of muscle fiber type in patients receiving EGFR inhibitor treatment for cancer would be valuable to determine whether EGFR inhibitors have this effect in humans *in vivo*, and therefore whether EGFR inhibitors may have therapeutic potential for patients, for example with COPD, with a locomotor muscle ST to FT fiber-type.

## Methods

### Pathway analysis

Data relating to differentially expressed genes in isolated mouse ST and FT IIb fibers taken from^18^ were analyzed through the use of IPA (QIAGEN Inc., https://www.qiagenbioinformatics.com/products/ingenuitypathway-analysis)^48^ to identify ST phenotype-associated pathways (see Supplementary Information).

### COPD patient and healthy control study

*Vastus lateralis* samples were collected for the study in^5^ which details clinical phenotyping, biopsy technique, and fiber typing. Studies were approved by the Royal Brompton and Harefield NHS Foundation Trust and Ealing and West London Mental Health Trust Research Ethics Committees (Studies 06/Q0404/35 and 06/Q0410/54) and all participants gave informed consent. Samples were stored in accordance with the Human Tissue Authority guidelines. The microarray analysis is described in^25^ and RNA extraction, cDNA synthesis and real-time quantitative PCR and quantification of serum EGF concentrations by ELISA are detailed in the Supplementary Information.

### Cell culture experiments

C2C12 mouse myoblasts^49^ were differentiated for 7 days into multinucleate myotubes, which co-express ST and FT myosins within single cells. From day 8 myotubes were treated daily for 3 days with 100 ng/ml EGF neutralizing antibody (EGFnAb) or 200 nM tyrphostin/AG-1478 or the respective vehicle control. 200 nM AG-1478 reportedly elicits preferential EGFR inhibition (IC_50_ of 3 nM compared to >100 uM for ErbB2 and PDGF receptors^50^ *in vitro*) and downstream MAPK/extracellular related kinase (ERK) signaling inhibition in different cells^51,52^. Myoblasts were also transfected with 1.5 μM siRNA to EGFR or scrambled negative control, differentiated for 3 days then lysed for RNA or stained with anti-MyHC I antibody (DSHB Cat# BA-F8, RRID: AB_10572253) or MitoTracker Red (ThermoFisher Scientific). qPCR was performed with HPRT as the housekeeping gene. (This approach was used because transfection efficiency of genetic material into fully differentiated myotubes is poor without electroporation^53^). Protein was extracted from myotubes treated as above and from myotubes treated with same AG-1478 and EGFnAb concentrations and lysed 1-hour post dose. Extracts were immunoblotted for phosphorylated EGFR (phosphorylated at tyrosine 1068, 175 kDa), and for markers of downstream EGFR signaling, pERK [pERK1 phosphorylated at Thr202 and/or Tyr204 (44 kDa), pERK2 phosphorylated at Thr185 and/or Tyr187 (42 kDa)] and pAkt (Akt 1, 2 and 3 phosphorylated at serine 473, 60 kDA), then stripped and re-probed for total proteins (for the 1-hour post dose samples). Details are in the Supplementary Information.

### Zebrafish experiments

Zebrafish on the AB background were housed and manipulated under UK Home Office Licence and standard procedures^54^. Whole-mount *in situ* hybridization for *egfra* was performed to confirm *egfra* expression as previously^55^. The CRISPR/Cas9 system was used to generate exon 1 *egfra* gene mutations and F3 generation allele number *kg134* heterozygotes and their WT siblings were analysed (see Supplementary Information for details). RNA and DNA were extracted from pools of 5 larvae at 2 days post-fertilisation (dpf) for qPCR (normalization to geomean of beta-2 microglobulin and TATA-binding protein transcripts) and from single larva for genotyping. F2 *egfra*^+/*kg134*^ males were crossed to tagged ß-actin: GFP Tg(Ola.Actb:Hsa.HRAS-EGFP)^*vu119*^ line^56^ females. Resulting GFP-positive *egfra*^+/*kg134*^ larvae and WT siblings were immunostained with anti-myosin antibody (DSHB Cat# A4.1025, RRID: AB_528356) at 2 dpf and ST fibers which exist at the outer somite border were counted in a standard somite sample (16–18) by an investigator blind to genotype.

### Statistical analysis

Exhaustive glm model comparison was carried out using the model selection and multimodel inference package *glmulti*, applying the Akaike information criterion. Continuous data was compared with a two-tailed t-test or Mann Whitney U-test depending on normality. Categorical data and correlations were analysed using Fisher’s exact test and calculation of Pearson’s coefficient respectively. A p-value < 0.05 was deemed statistically significant.

## Supplementary information

Supplementary information including figures

Supplementary Information - Full Blots for All Figures

Supplementary Video E12 of wild-type zebrafish sibling

Supplementary Video E12 of egfra +kg134 zebrafish sibling

Supplementary information excluding figures
