# Supplementary material for: EGF receptor (EGFR) inhibition promotes a slow-twitch oxidative, over a fast-twitch, muscle phenotype
Source: Sci Rep. 2019 Jun 25;9:9218. doi: 10.1038/s41598-019-45567-4 (PMC6592914; doi:10.1038/s41598-019-45567-4)
Supplement: Supplementary file 1 — Supplementary information including figures [file 41598_2019_45567_MOESM1_ESM.pdf]

SUPPLEMENTARY INFORMATION INCLUDING FIGURES

FOR SREP 18-31535 R1

EGF receptor (EGFR) inhibition promotes a slow-twitch oxidative, over a fast-twitch, muscle phenotype

Margherita Ciano[1], Giada Mantellato[2], Martin Connolly[2], Mark Paul-Clark[2], Saffron Willis-Owen[2], Miriam F Moffatt[2], William O.C.M. Cookson[2], Jane A Mitchell[2], Michael I Polkey[3], Simon M. Hughes[4], Paul R. Kemp[2], S. Amanda Natanek[2].

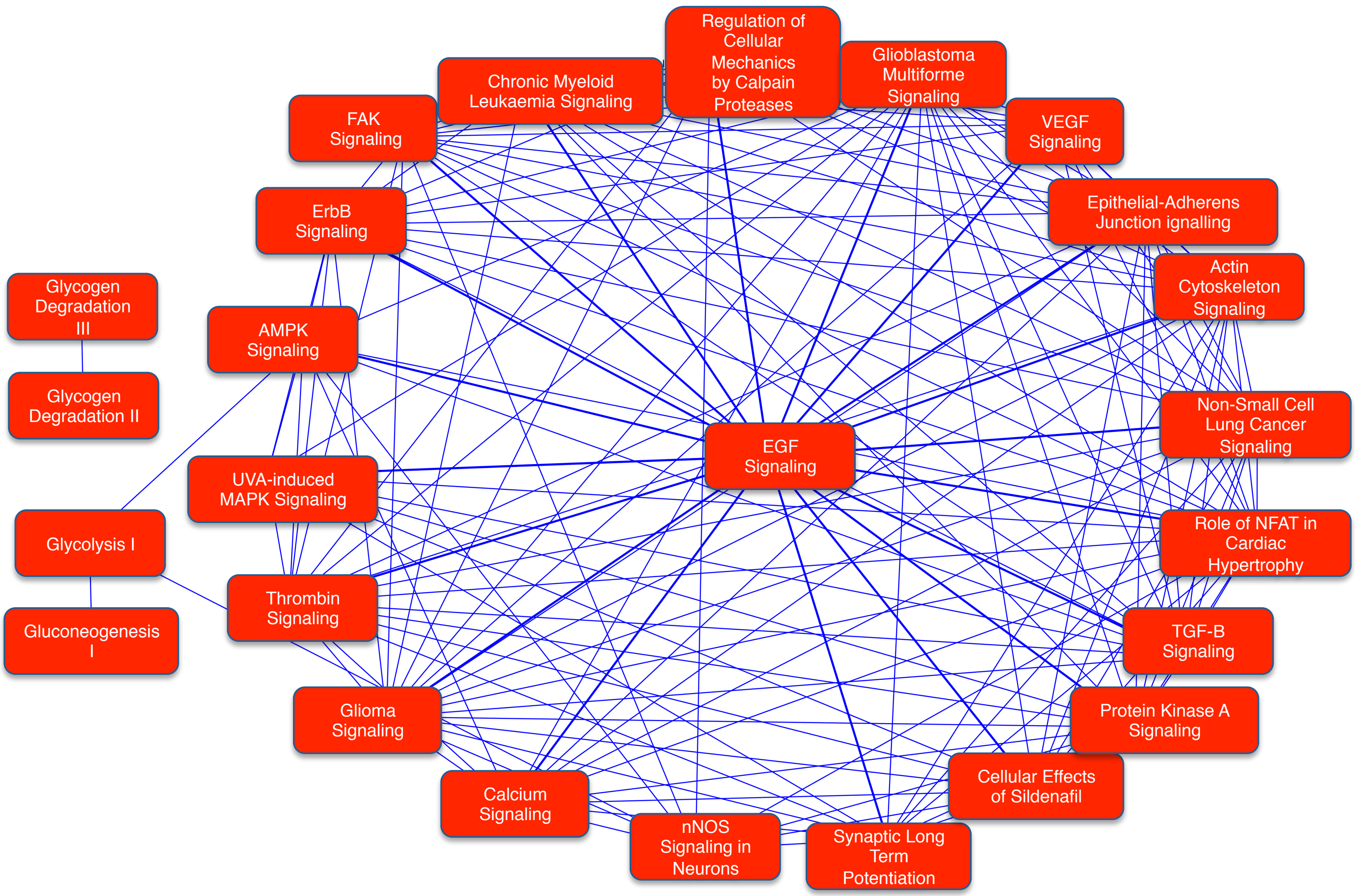

Figure E1 Network driven by EGF signaling associated with repression of ST muscle phenotype identified by Ingenuity Pathway Analysis of published mouse data

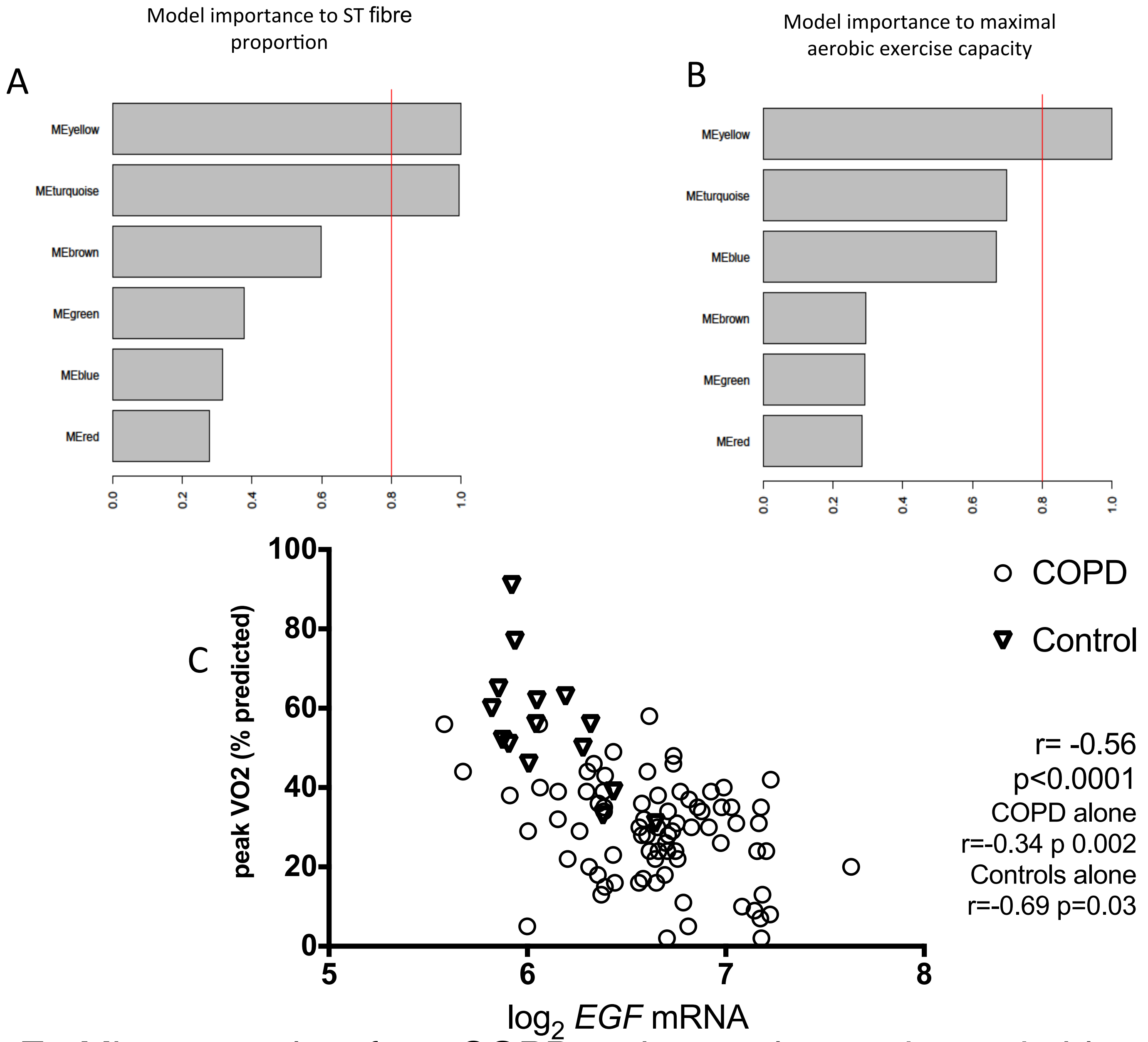

Figure E2 Microarray data from COPD patient and control muscle identifying the module contribution to ST fibre proportion (A) and exercise capacity, and the relationship between expression of EGF (within the yellow module) and exercise capacity

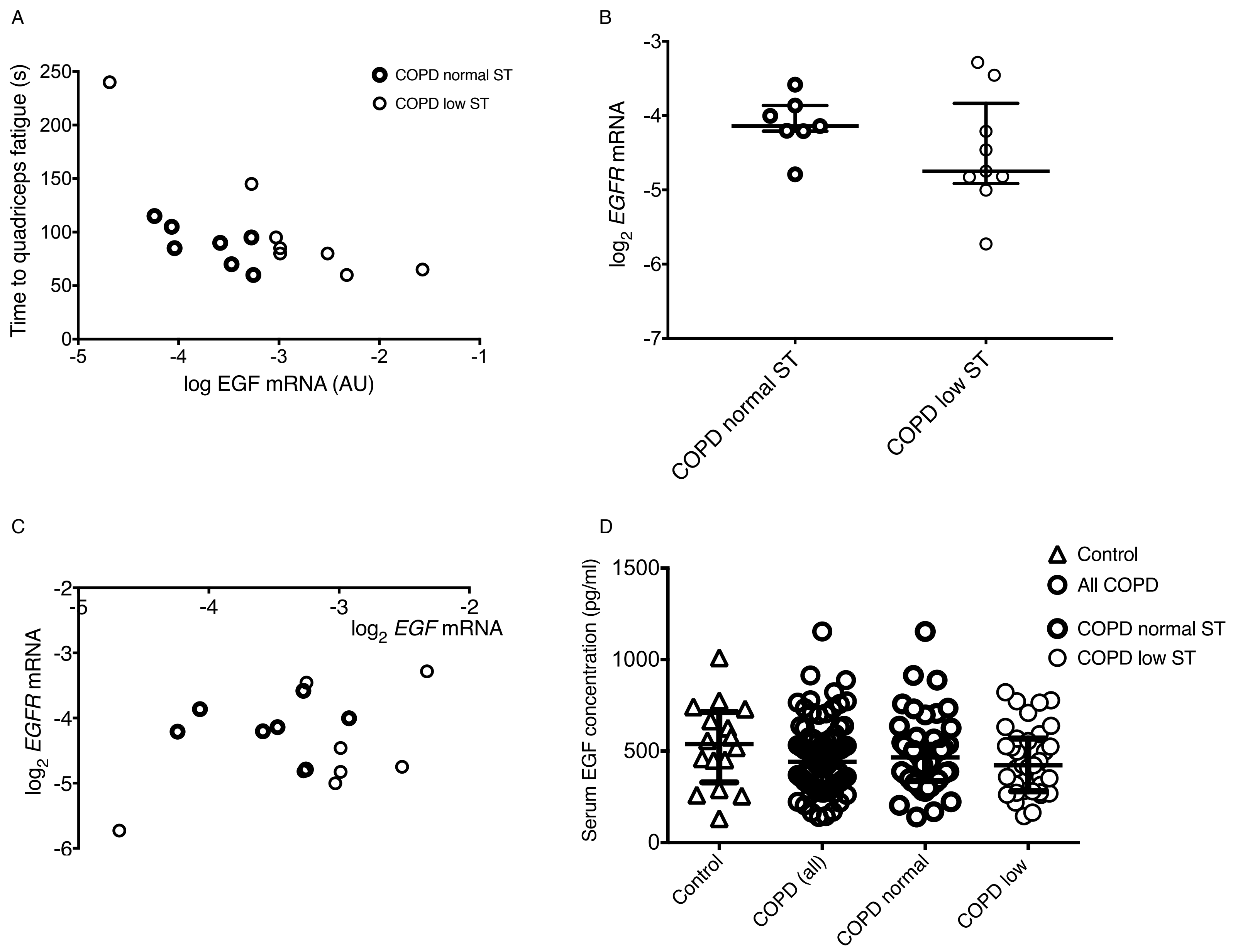

Figure 3 *EGF* and *EGFR* transcripts in quadriceps muscle of COPD patients in small PCR cohort and serum EGF protein concentrations in large patient and control cohort

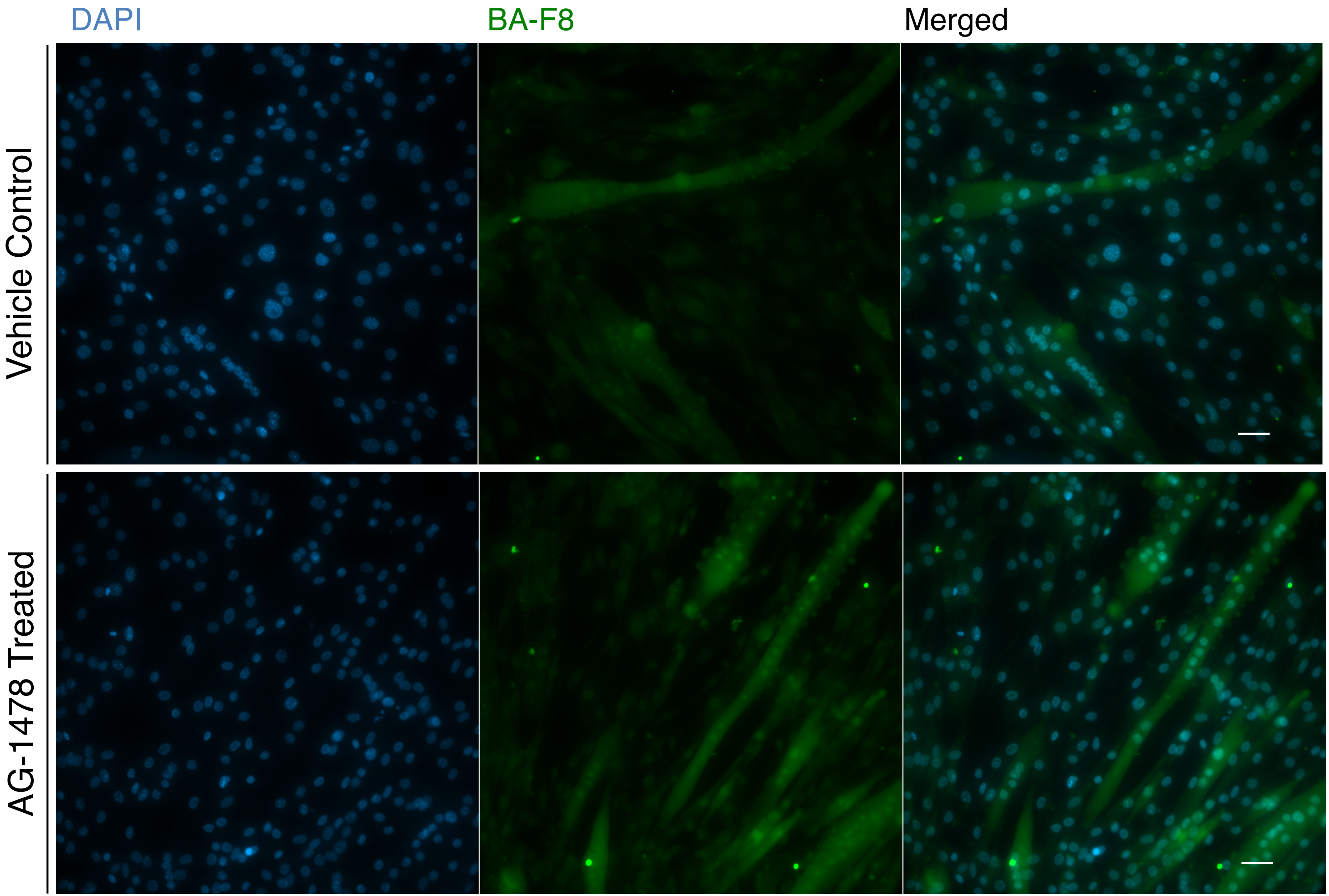

Figure E4 Representative images of MyHC I and nuclear staining in C2C12 myotubes treated with AG-1478 or vehicle control

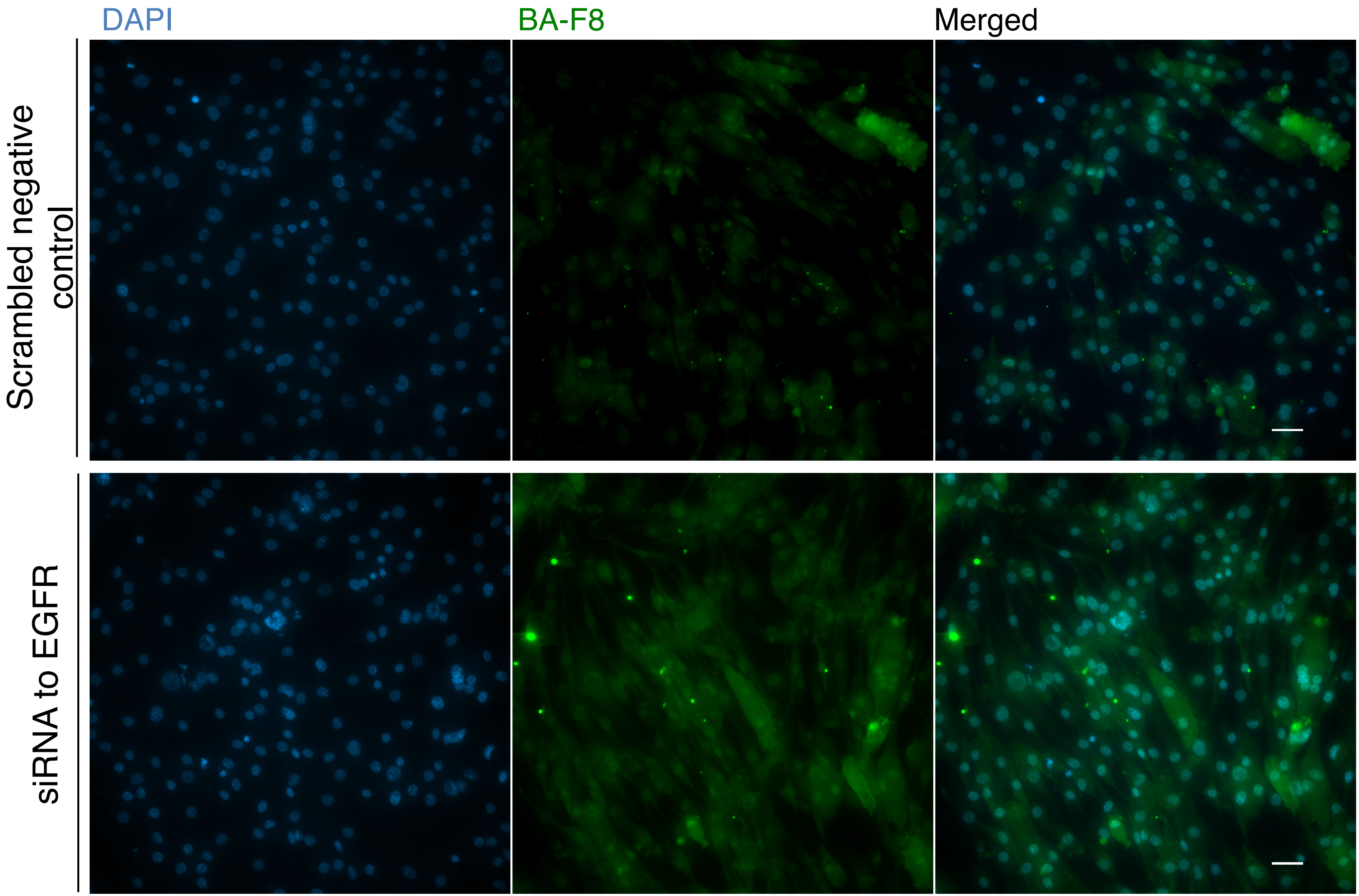

Figure E5 Representative images of MyHC I and nuclear staining in C2C12 myotubes treated with siRNA to the EGFR or scrambled negative control

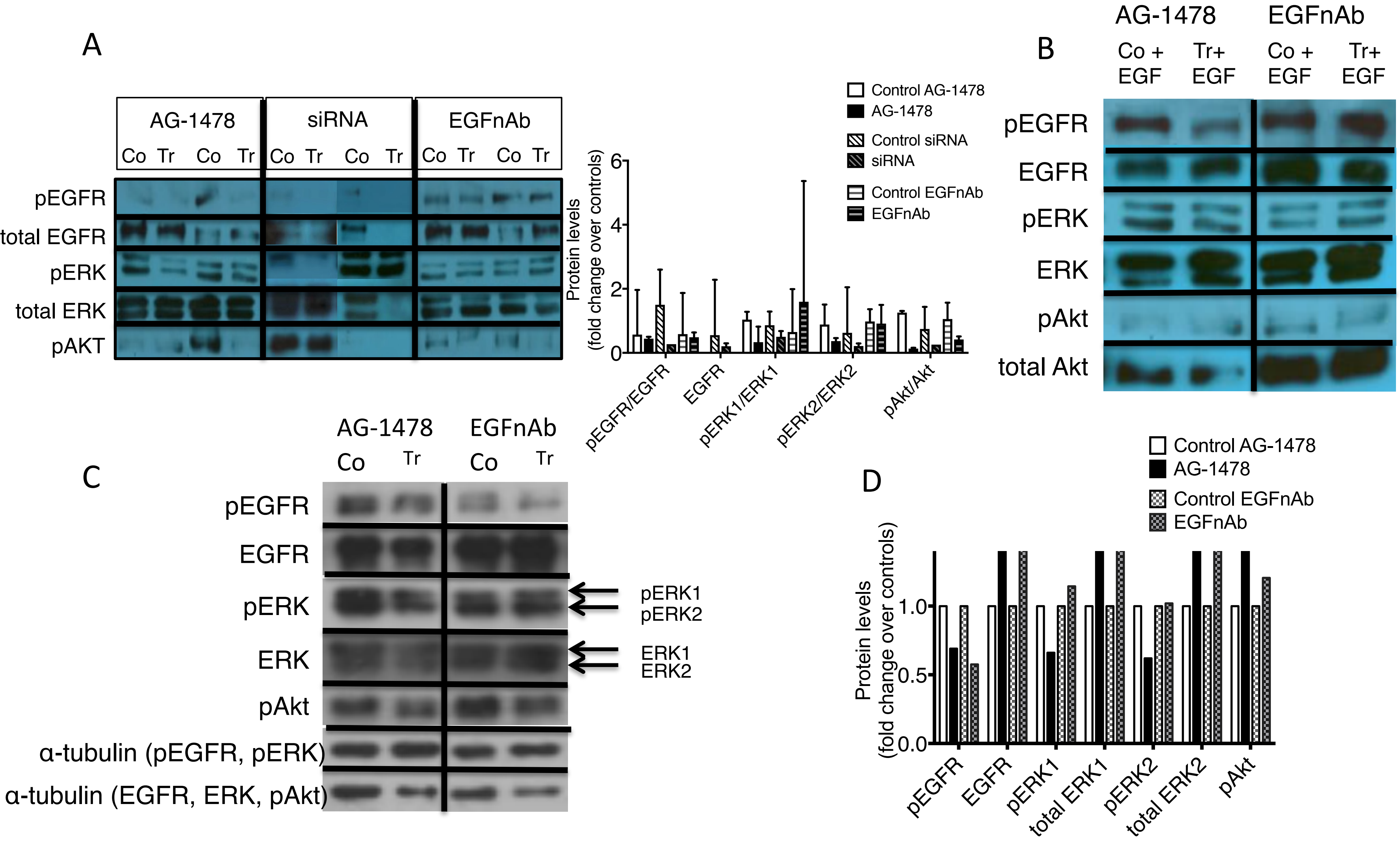

Figure E6 Western blot image of proteins extracted from C2C12 myotubes (n=2) and quantification of protein bands (n=3) 1 hour after AG1478 or EGF neutralizing antibody treatment or 3 days after transfection of EGFR siRNA or respective vehicle control (Fig A),1 hour after AG1478 or EGF neutralizing antibody treatment or respective vehicle control plus 100ng/ml recombinant EGF added to all conditions (Fig B), 24 hours after final treatment with AG1478, EGF neutralizing antibody or respective vehicle control (Fig C) and graph of quantification of bands in C (Fig D)

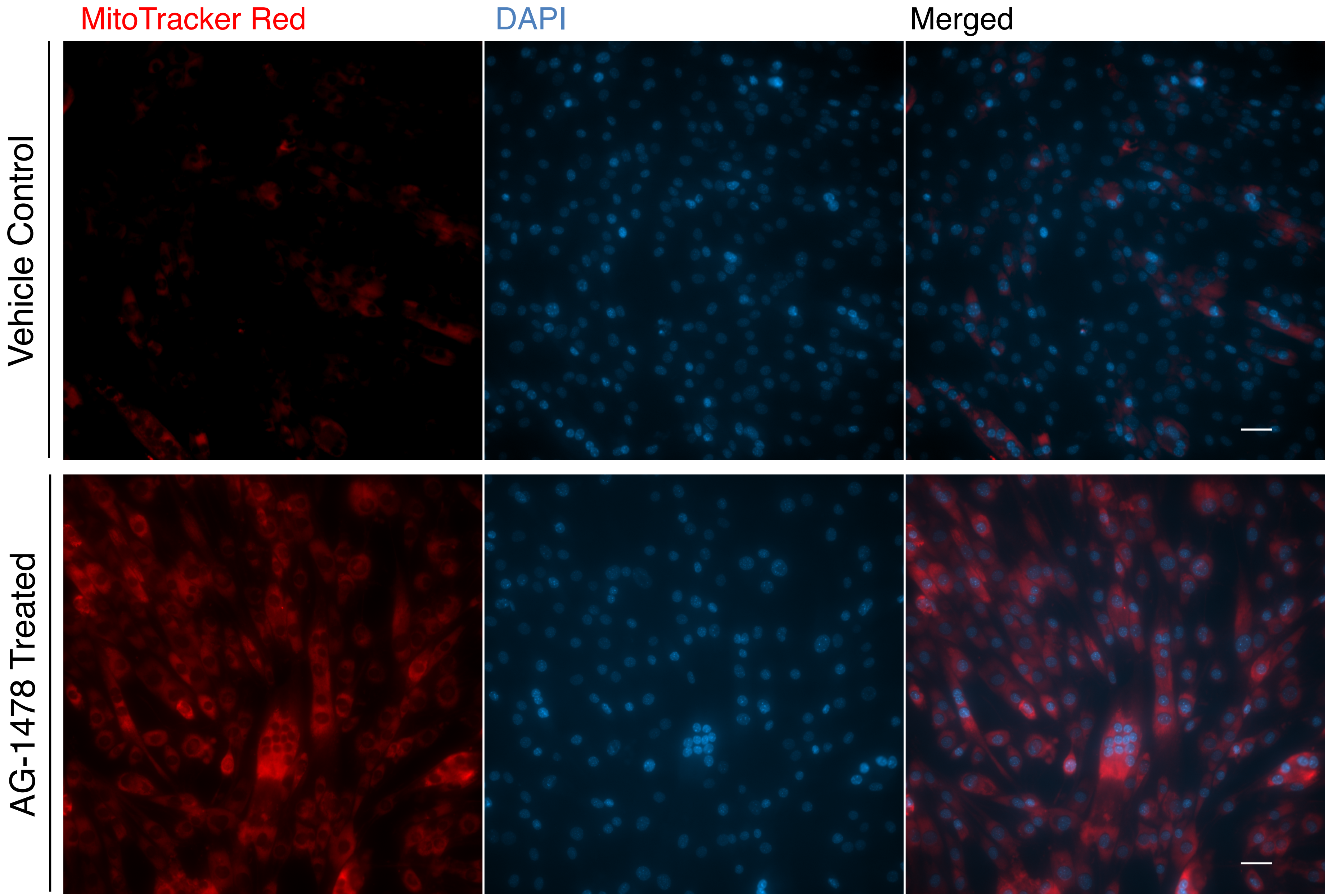

Figure E7 Representative images of MitoTracker® Red and nuclear staining of C2C12 myotubes treated with AG-1478 or vehicle control

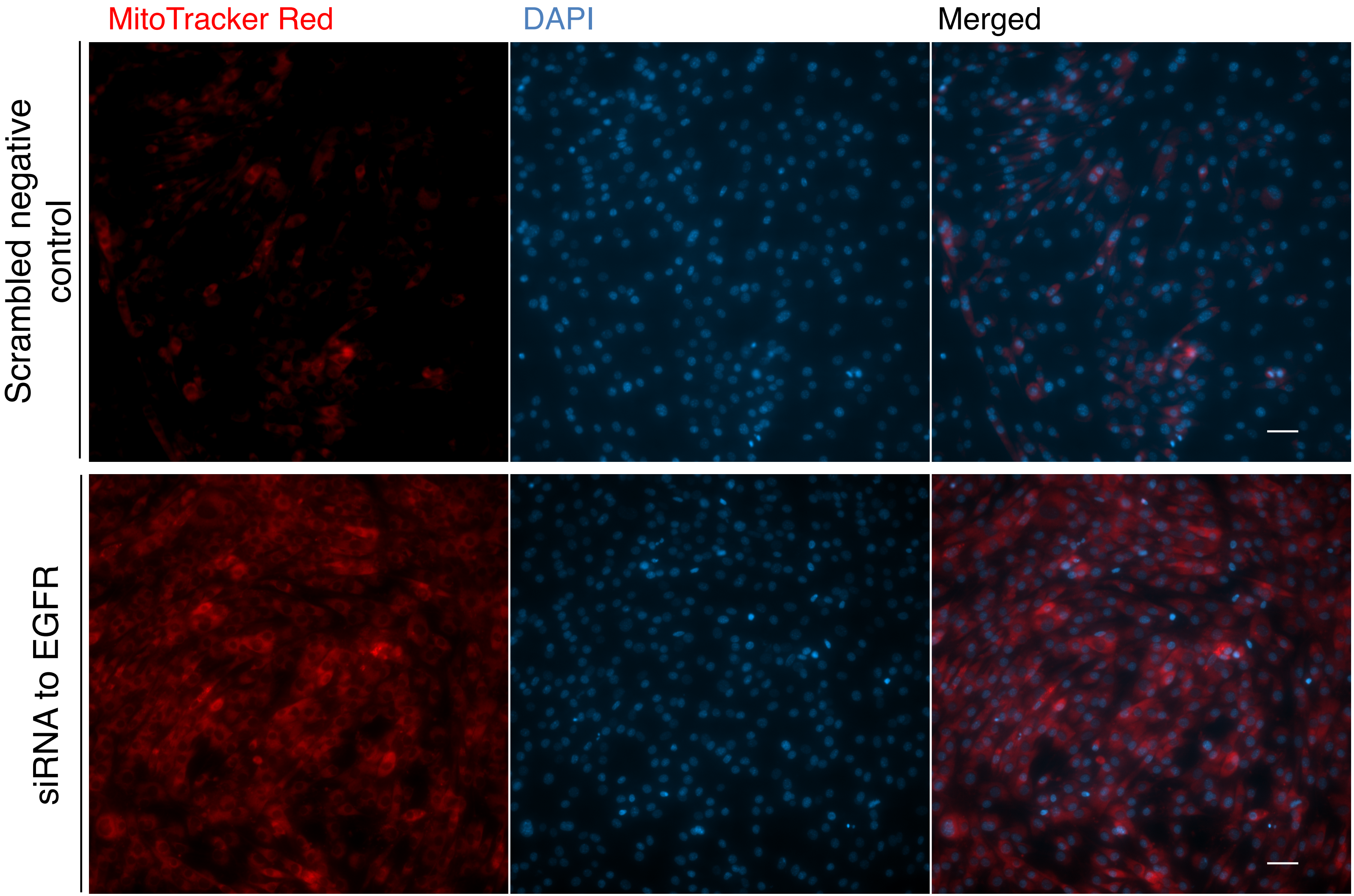

Figure E8 Representative images of MitoTracker® Red and nuclear staining C2C12 myotubes treated with siRNA to the EGFR or scrambled negative control

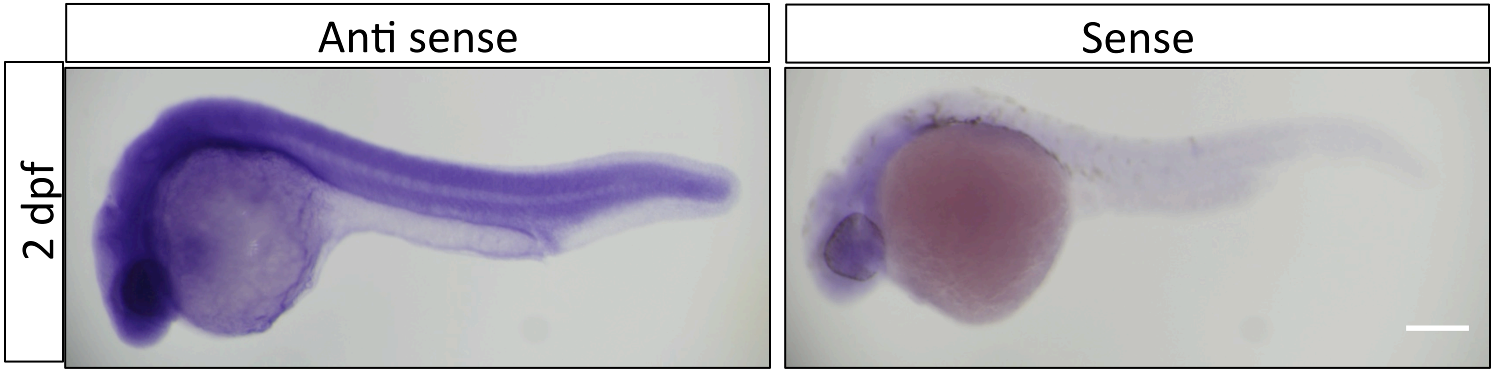

Figure E9 In situ mRNA hybridization for *egfra* transcripts in 2 dpf zebrafish larva showing no spatially restricted expression

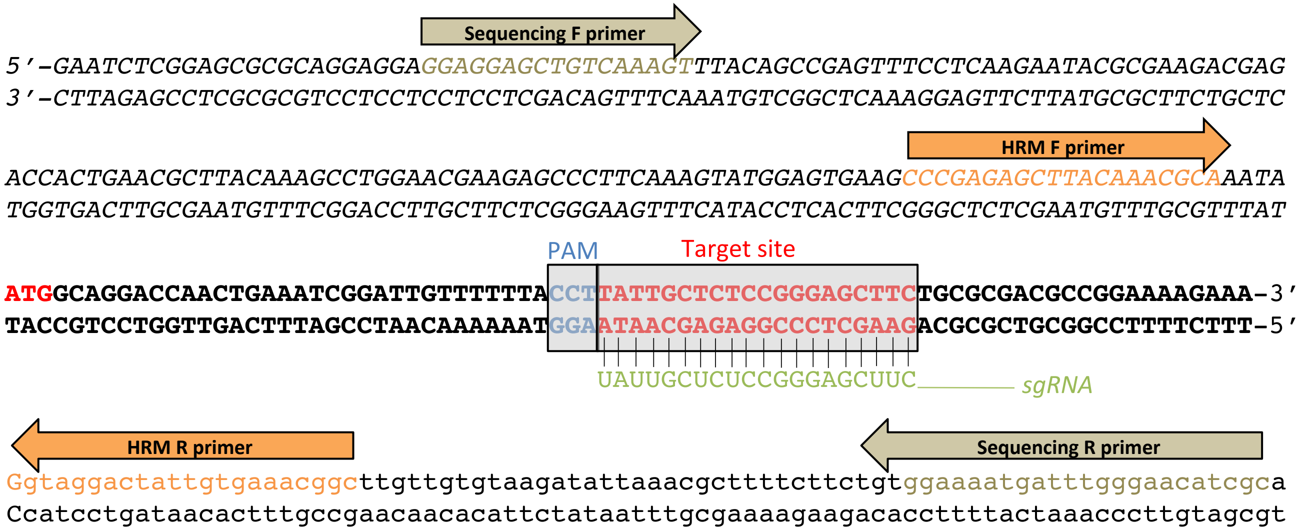

Figure E10 Schematic of initial nucleotide sequence of Exon 1 of the *egfra* gene in *Danio rerio* showing the region targeted by the single guide RNA

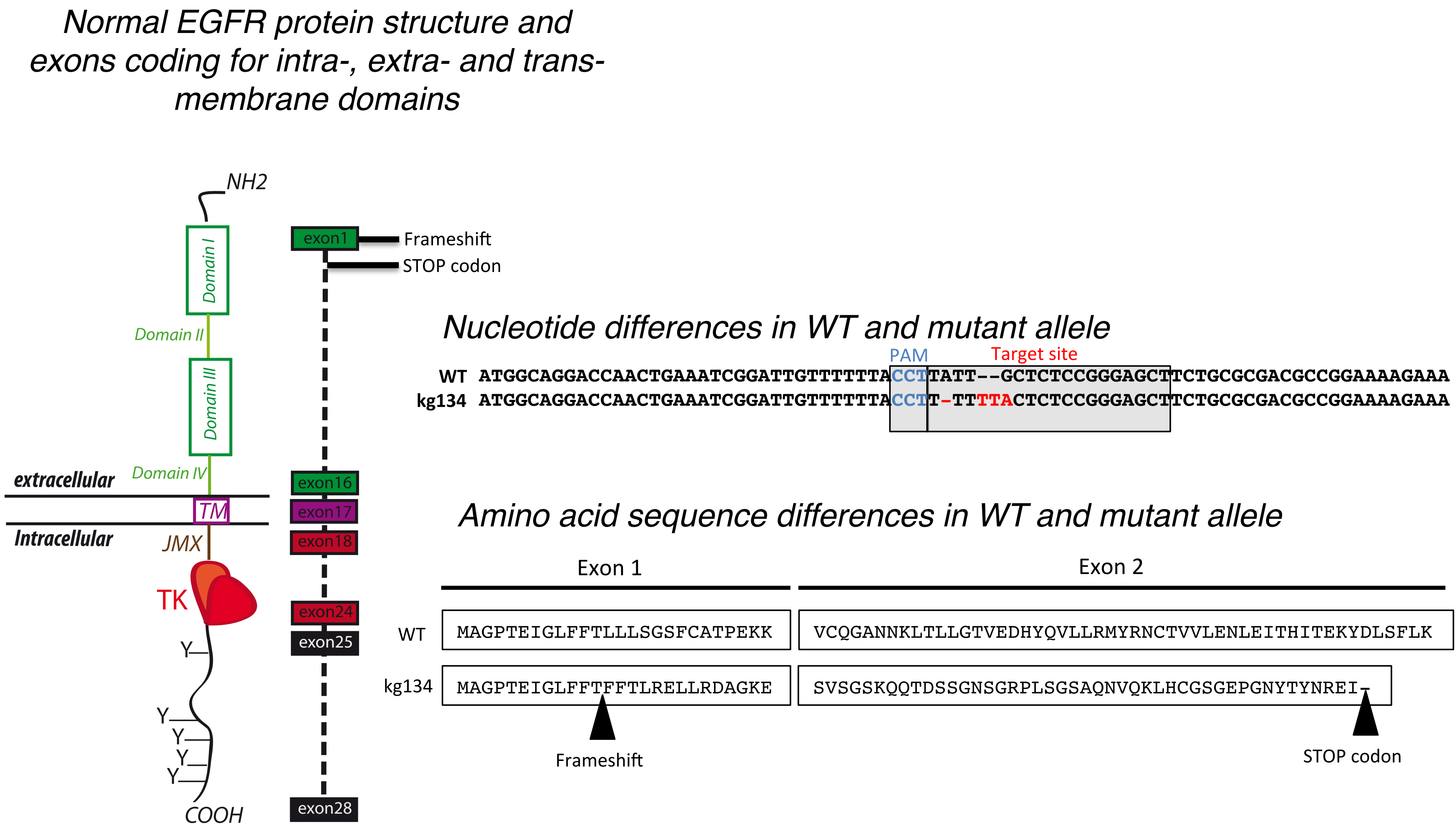

Figure E11 Schematic of mutation and frameshift in exon 1 of *egfra* and truncated peptide sequence in the *egfra*$^{+/-kg134}$ zebrafish with reference to the normal EGFR protein structure
