# Supplementary material for: EGF receptor (EGFR) inhibition promotes a slow-twitch oxidative, over a fast-twitch, muscle phenotype
Source: Sci Rep. 2019 Jun 25;9:9218. doi: 10.1038/s41598-019-45567-4 (PMC6592914; doi:10.1038/s41598-019-45567-4)
Supplement: Supplementary file 2 — Supplementary Information - Full Blots for All Figures [file 41598_2019_45567_MOESM2_ESM.pdf]

EGF receptor (EGFR) inhibition promotes a slow-twitch oxidative, over a fast-twitch, muscle phenotype

Margherita Ciano, Giada Mantellato, Martin Connolly, Mark Paul-Clark, Saffron Willis-Owen, Miriam F Moffatt, William O.C.M. Cookson, Jane A Mitchell, Michael I Polkey, Simon M. Hughes, Paul R. Kemp, S. Amanda Natanek

Corresponding author:

Dr Samantha Amanda Natanek, Molecular Medicine, NHLI, SAF Building, Imperial College London,
London, SW7 2AZ, UK. +44 (0)20 75
94 2831. a.natanek@imperial.ac.uk

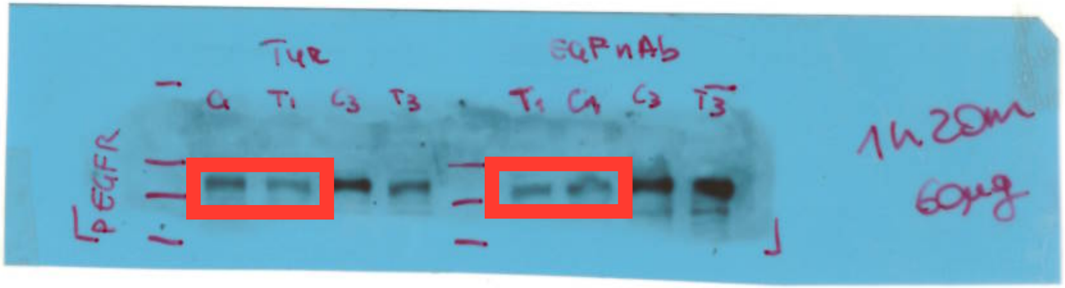

Tyr
EGFnAb
C1 T1 C3 T3
T1 C1 C3 T3
pEGFR
1h20m
60µg

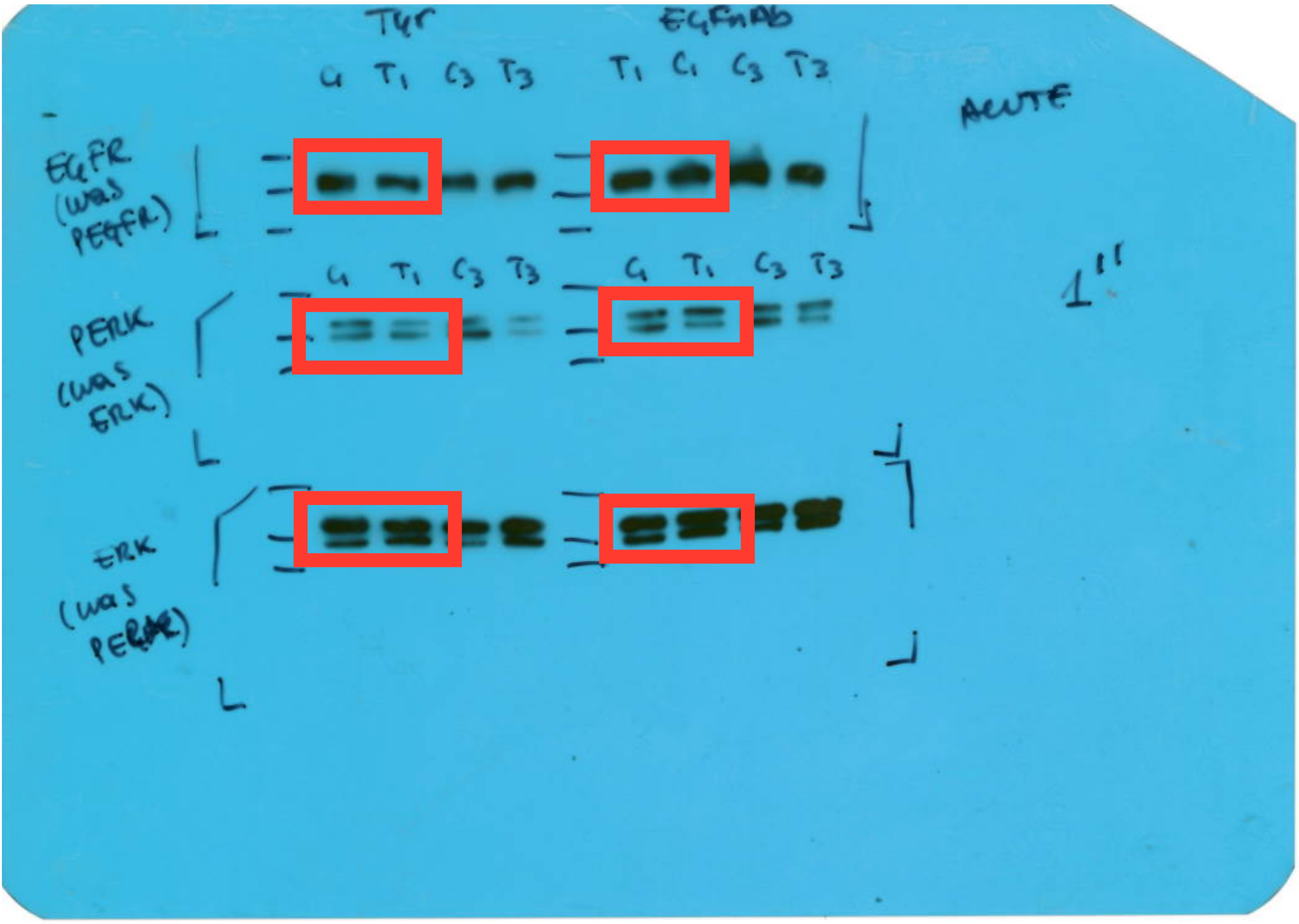
Tyr
EGFmAb
C1 T1 C3 T3
T1 C1 C3 T3
ACUTE
EGFR (was PEGFR)
PERK (was ERK)
ERK (was PERK)
1''

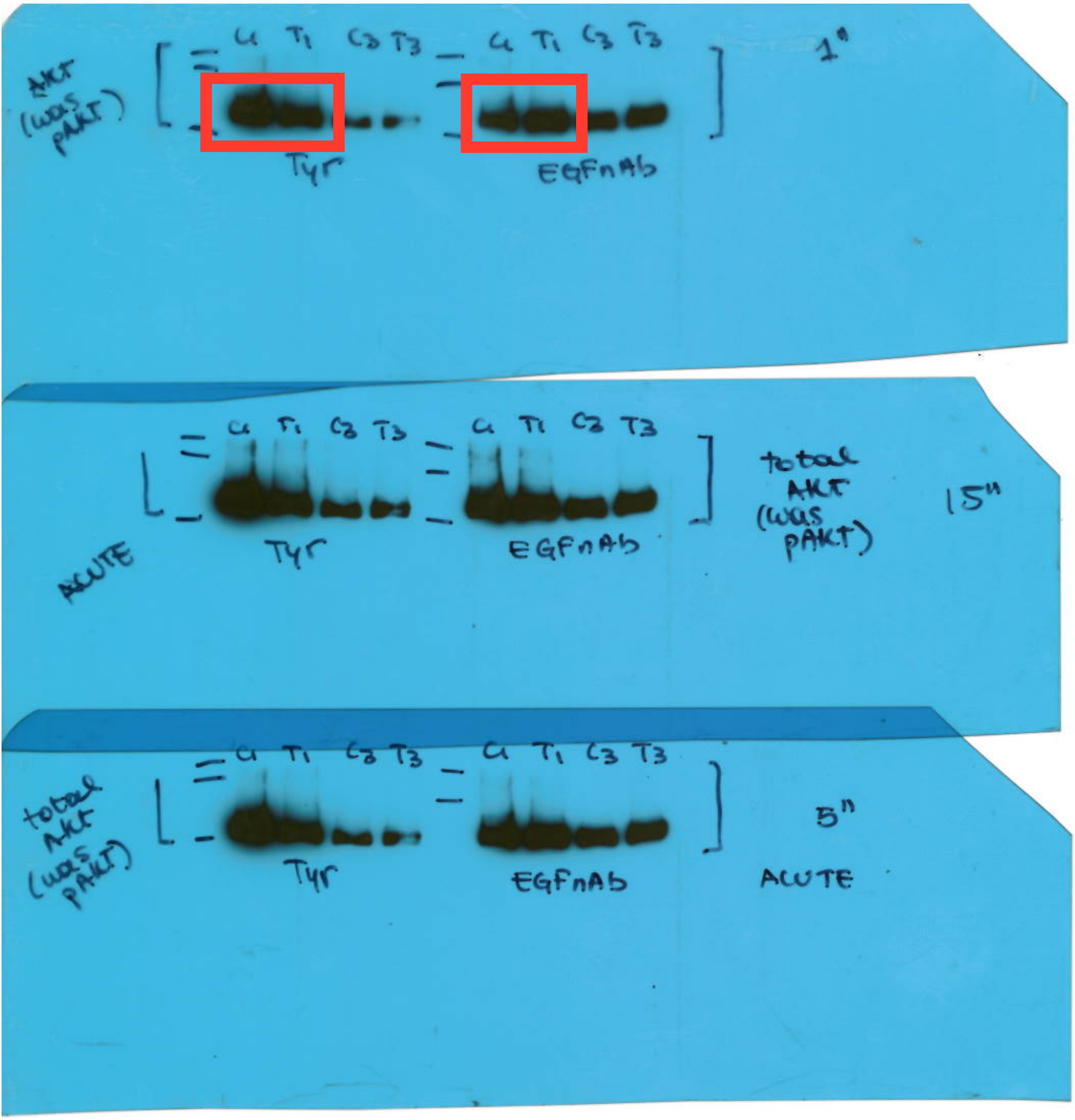
AKT (was pAKT)
C1 T1 C3 T3
C1 T1 C3 T3
1"
Tyr
EGFnAb
C1 T1 C3 T3
C1 T1 C3 T3
total AKT (was pAKT)
15"
Tyr
EGFnAb
ACUTE
total AKT (was pAKT)
C1 T1 C3 T3
C1 T1 C3 T3
5"
Tyr
EGFnAb
ACUTE

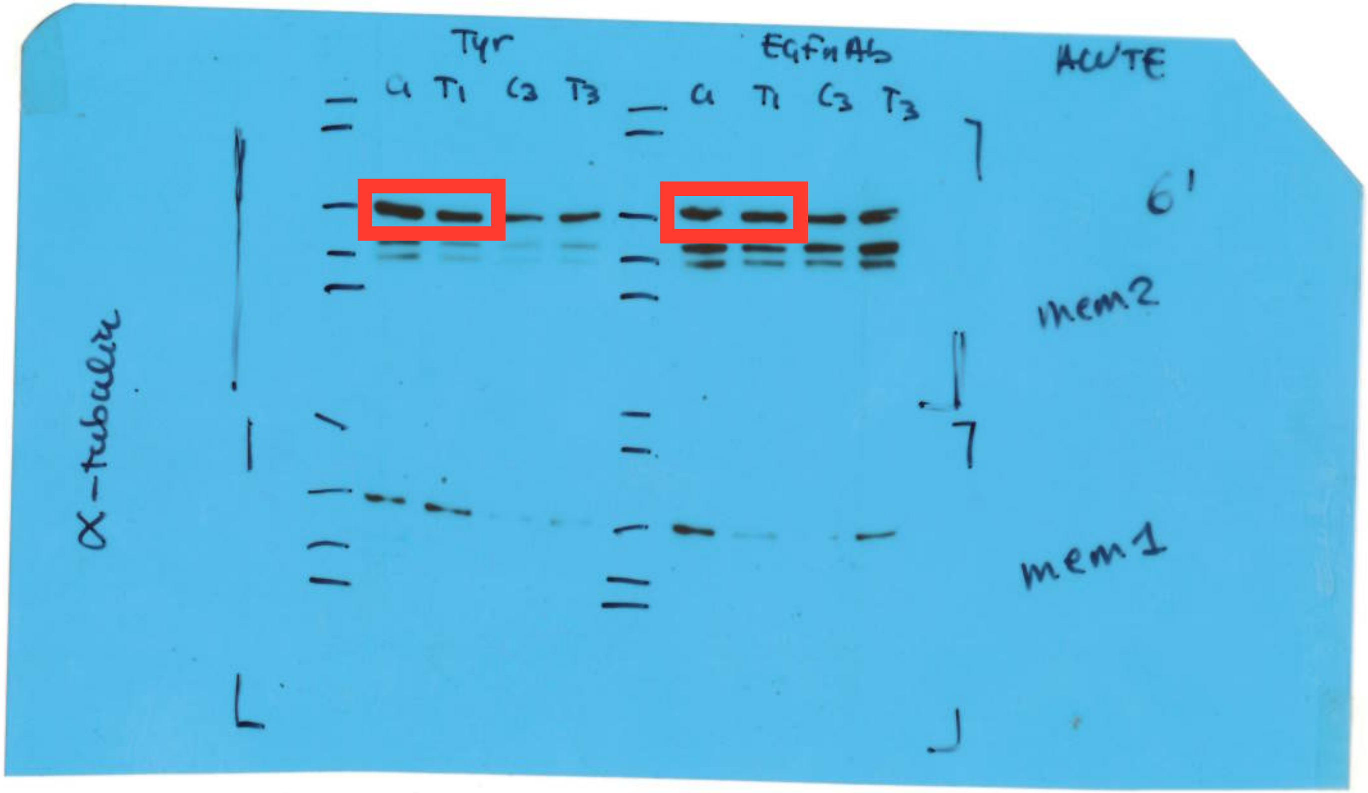
Tyr
C1 T1 C3 T3
C1 T1 C3 T3
ACUTE
6'
mem2
mem1
α-tubulin

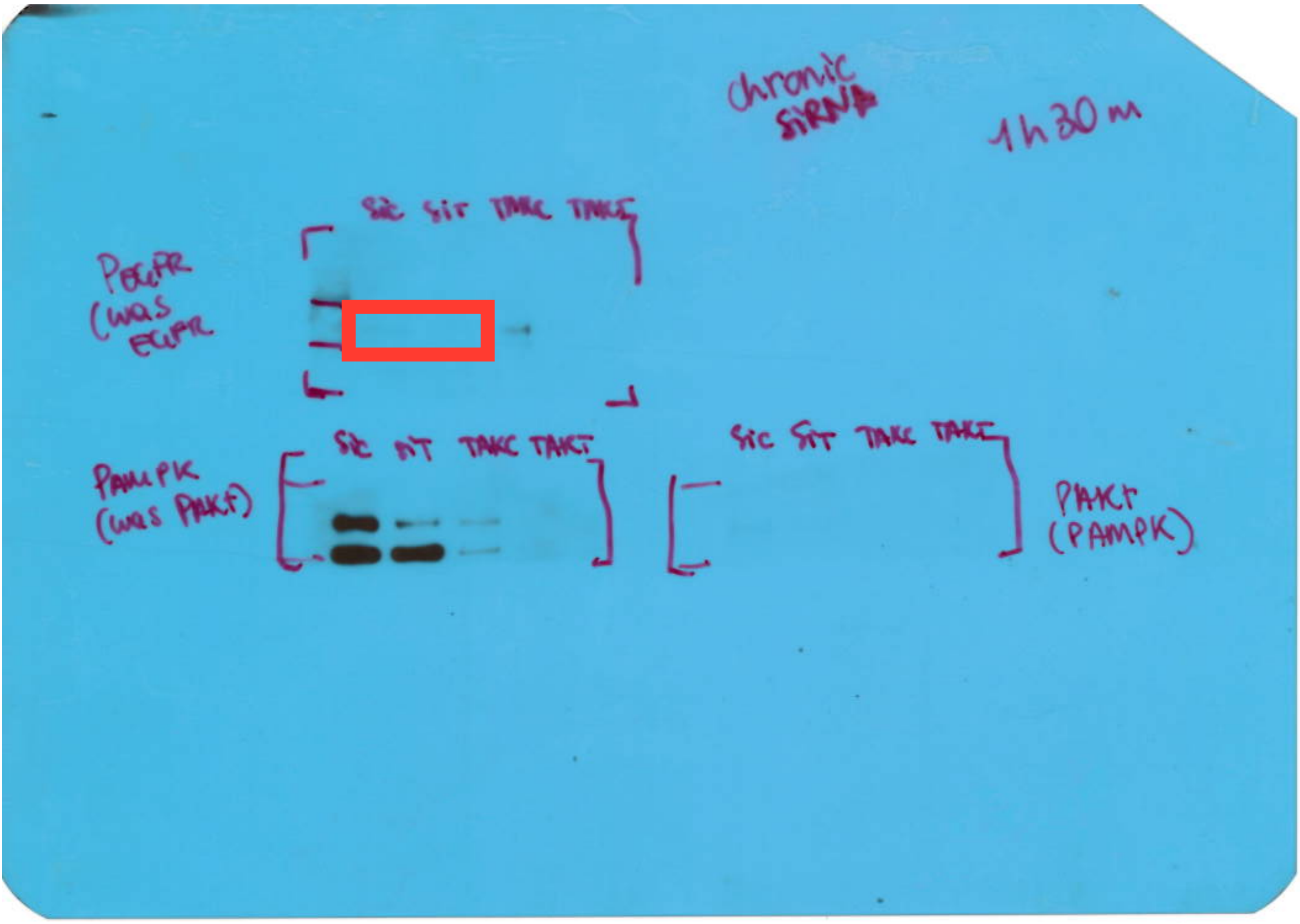
chronic
siRNA
1h30m
Sic SiT TAKC TAKT
PEGFR
(was
EGFR
Sic NT TAKC TAKT
PAMPK
(was PAKT)
Sic SiT TAKC TAKT
PAKT
(PAMPK)

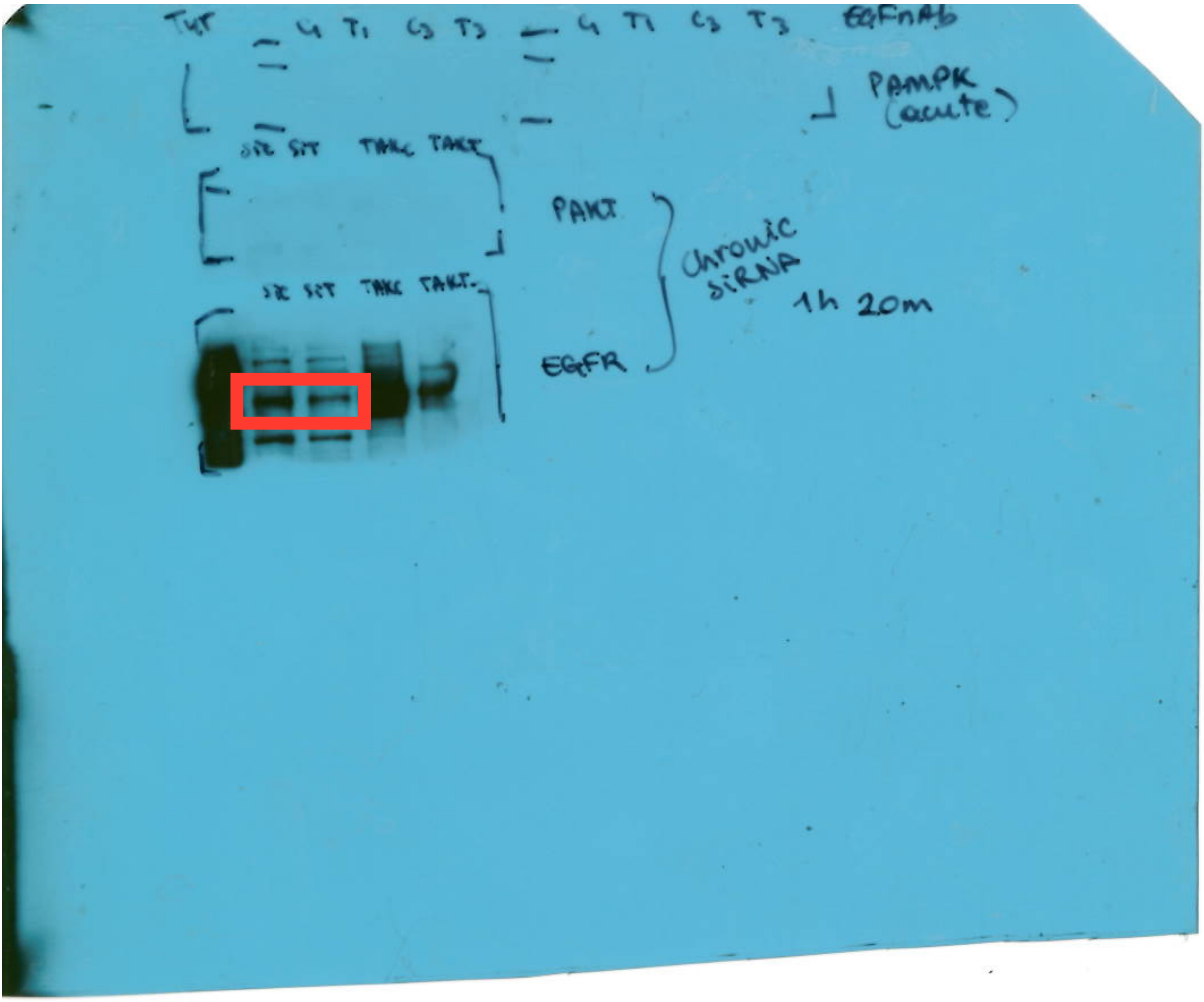

C1 T1 C3 T3
C1 T1 C3 T3
PAMPK (acute)
siC siT TAKC TAKT
PAKT
Chronic siRNA
1h 20m
siC siT TAKC TAKT
EGFR

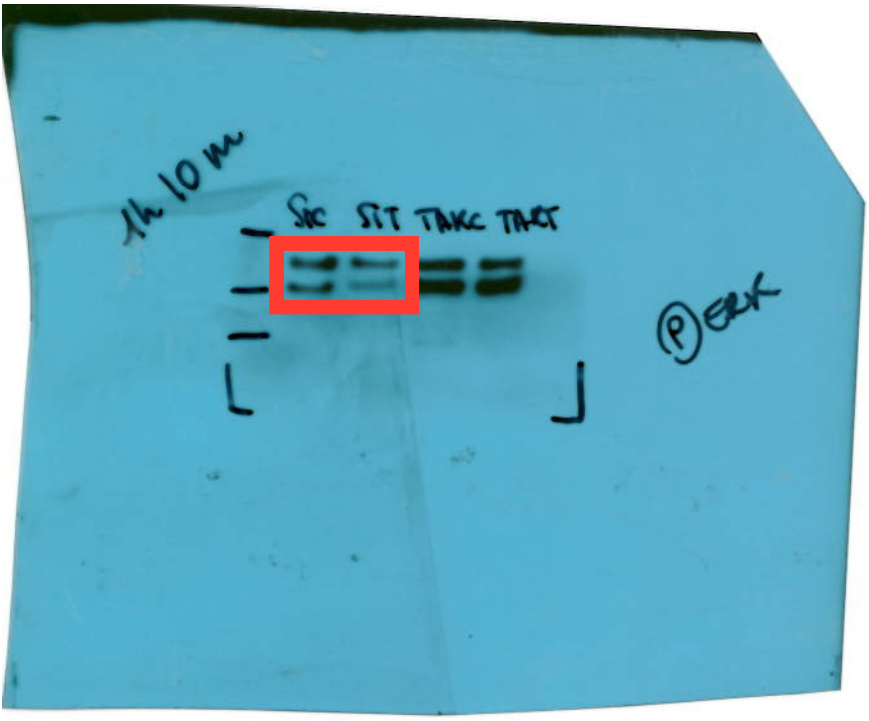
1h 10 m
SiC SiT TAKC TAKT
(P)ERK

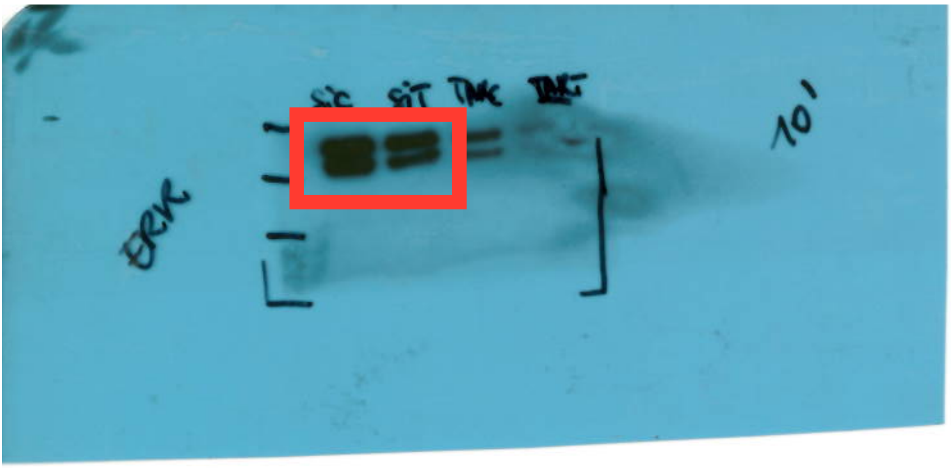
ERK
10'

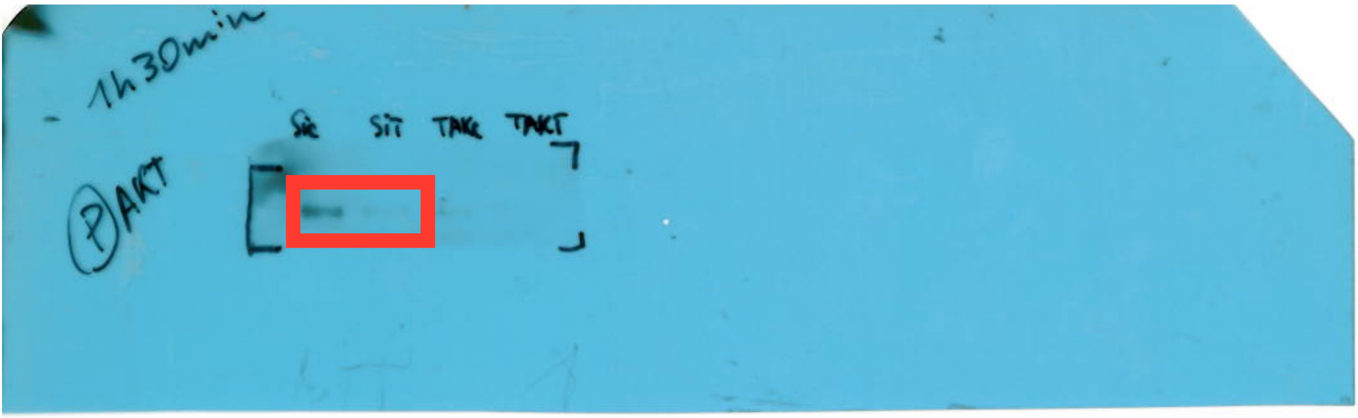
1h30min
Ⓟ AKT
Sic
SiT
TAKc
TAKT

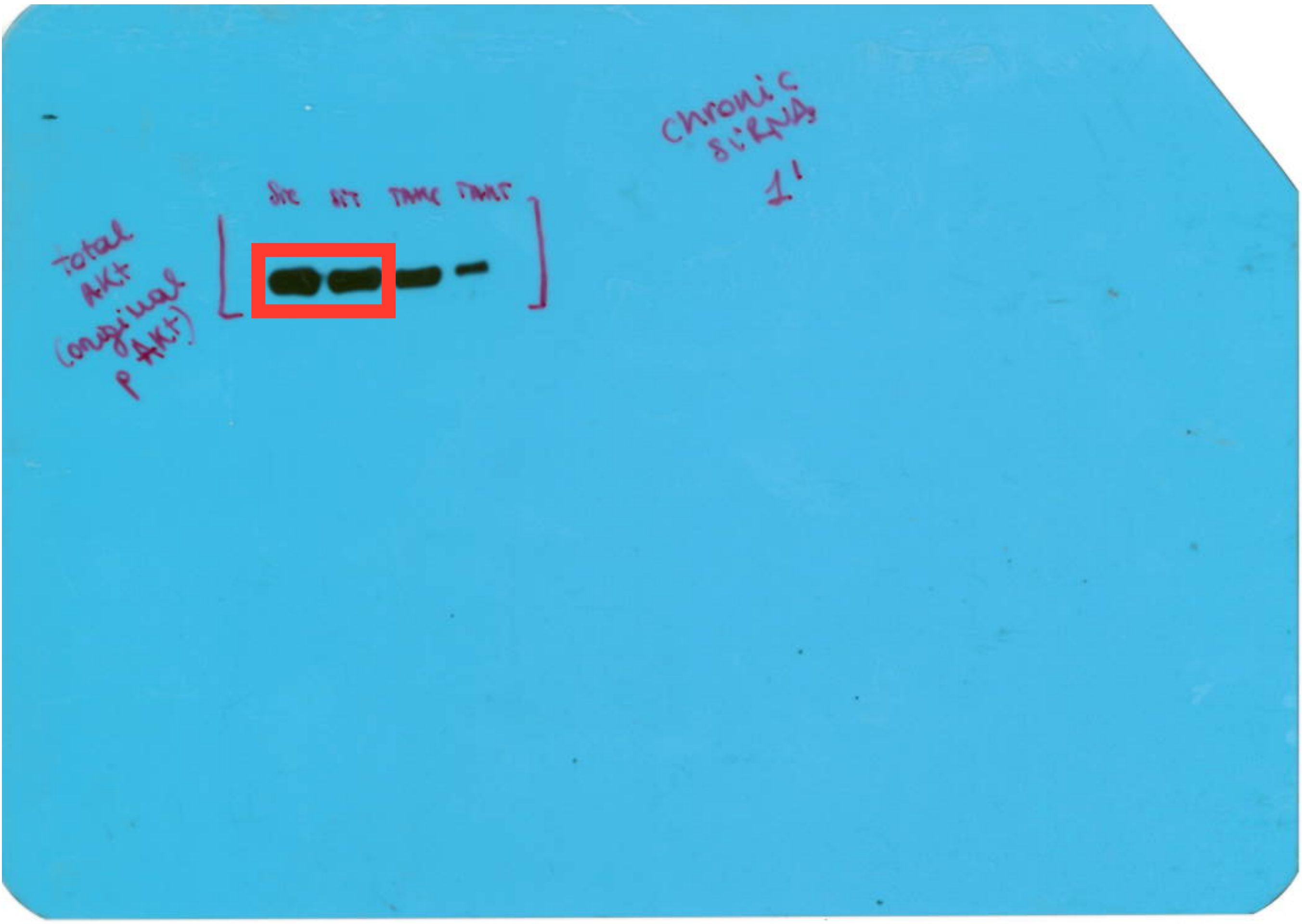
Total AKt (original p AKt)
Chronic siRNA 1'

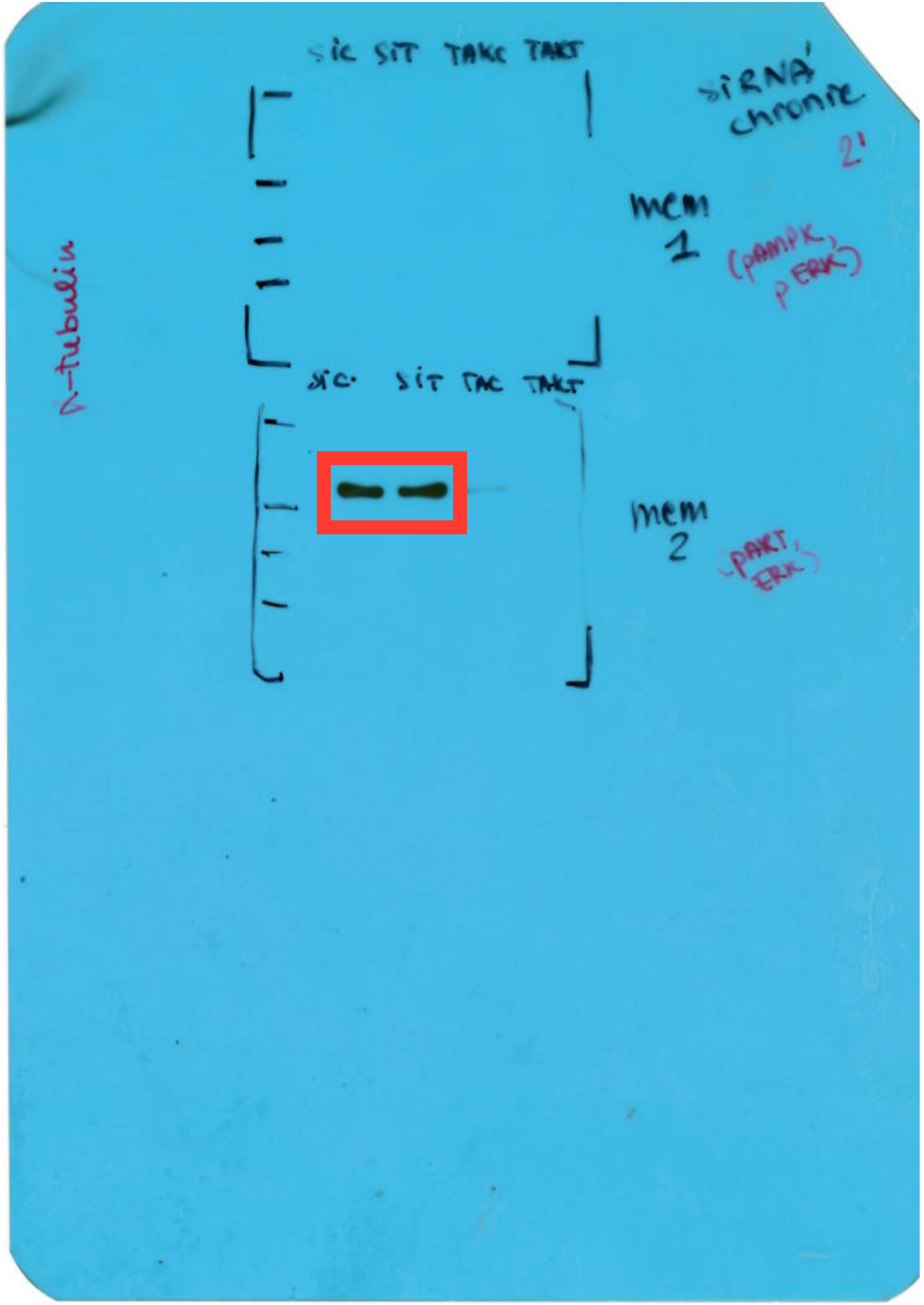
siC siT TAKC TAKT
siRNA chronic
mem 1
(pAMPK, pERK)
siC siT TAC TAKT
mem 2
(pAKT, ERK)

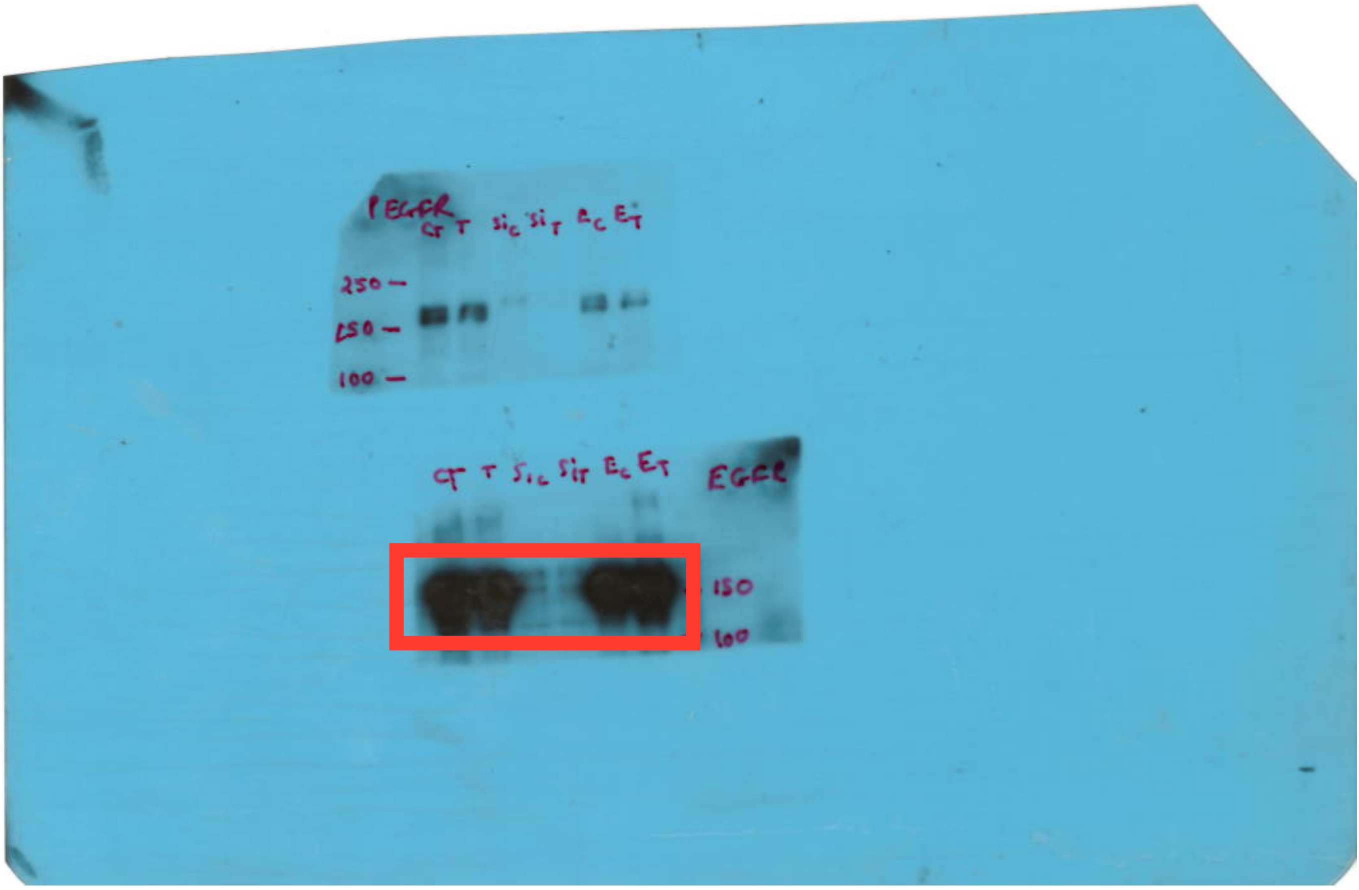

pEGFR
250 –
150 –
100 –
EGFR
150
100

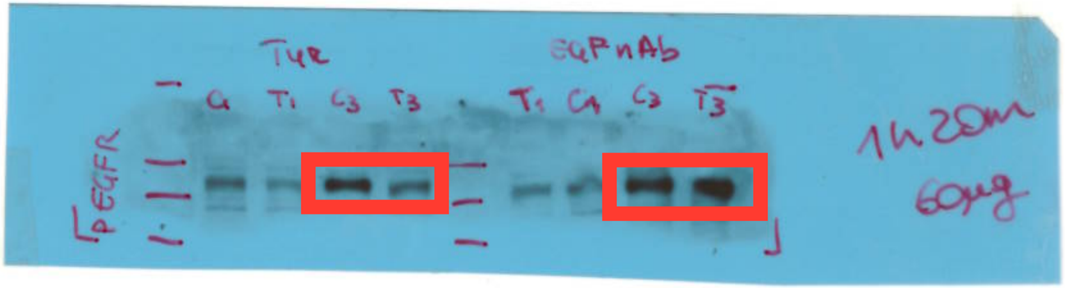
Tyr
EGFnAb
C1 T1 C3 T3
T1 C1 C3 T3
pEGFR
1h20m
60µg

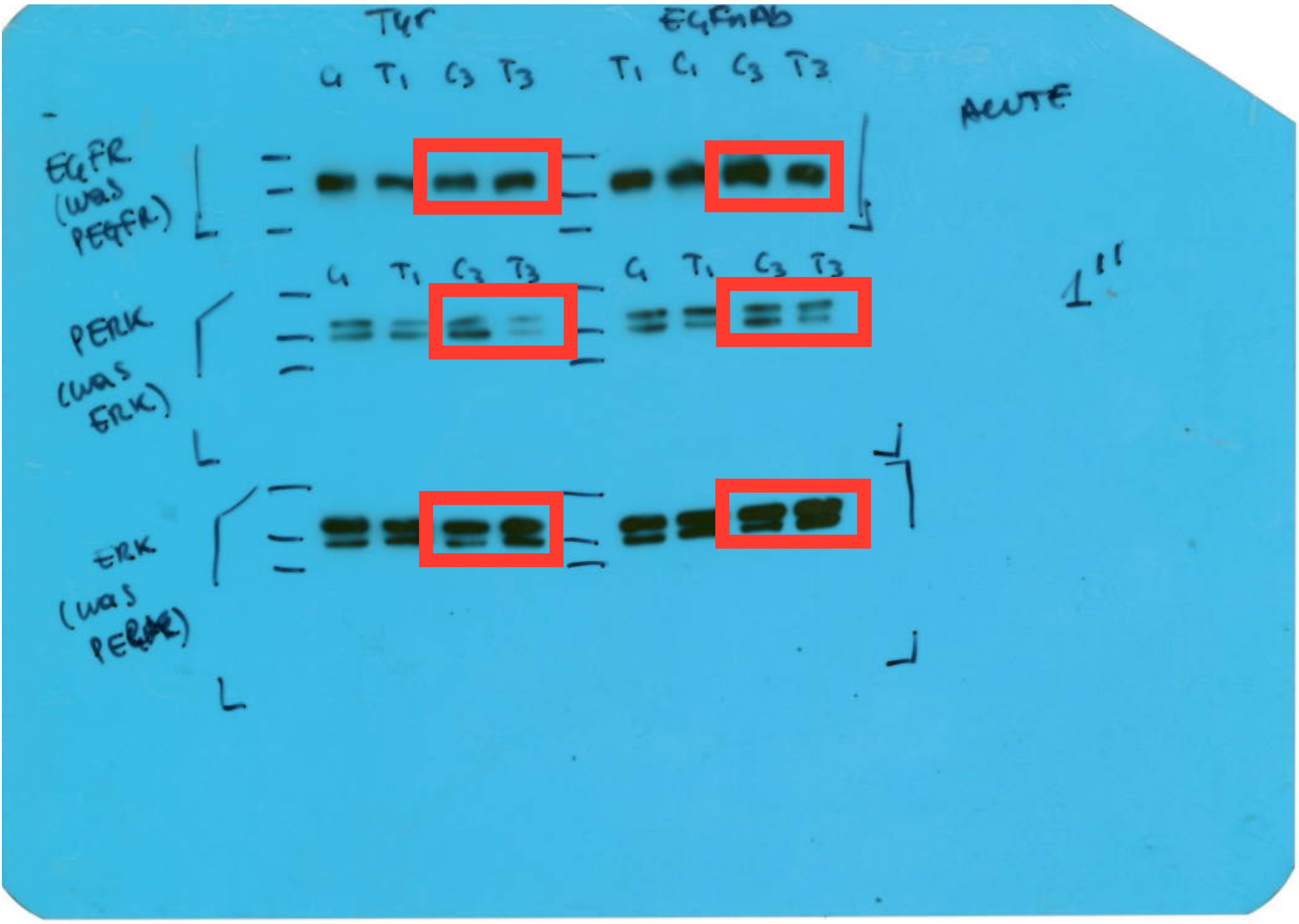
Tyr
EGFrAb
C1 T1 C3 T3
T1 C1 C3 T3
ACUTE
EGFR (was PEGFR)
C1 T1 C3 T3
C1 T1 C3 T3
PERK (was ERK)
ERK (was PERK)
1''

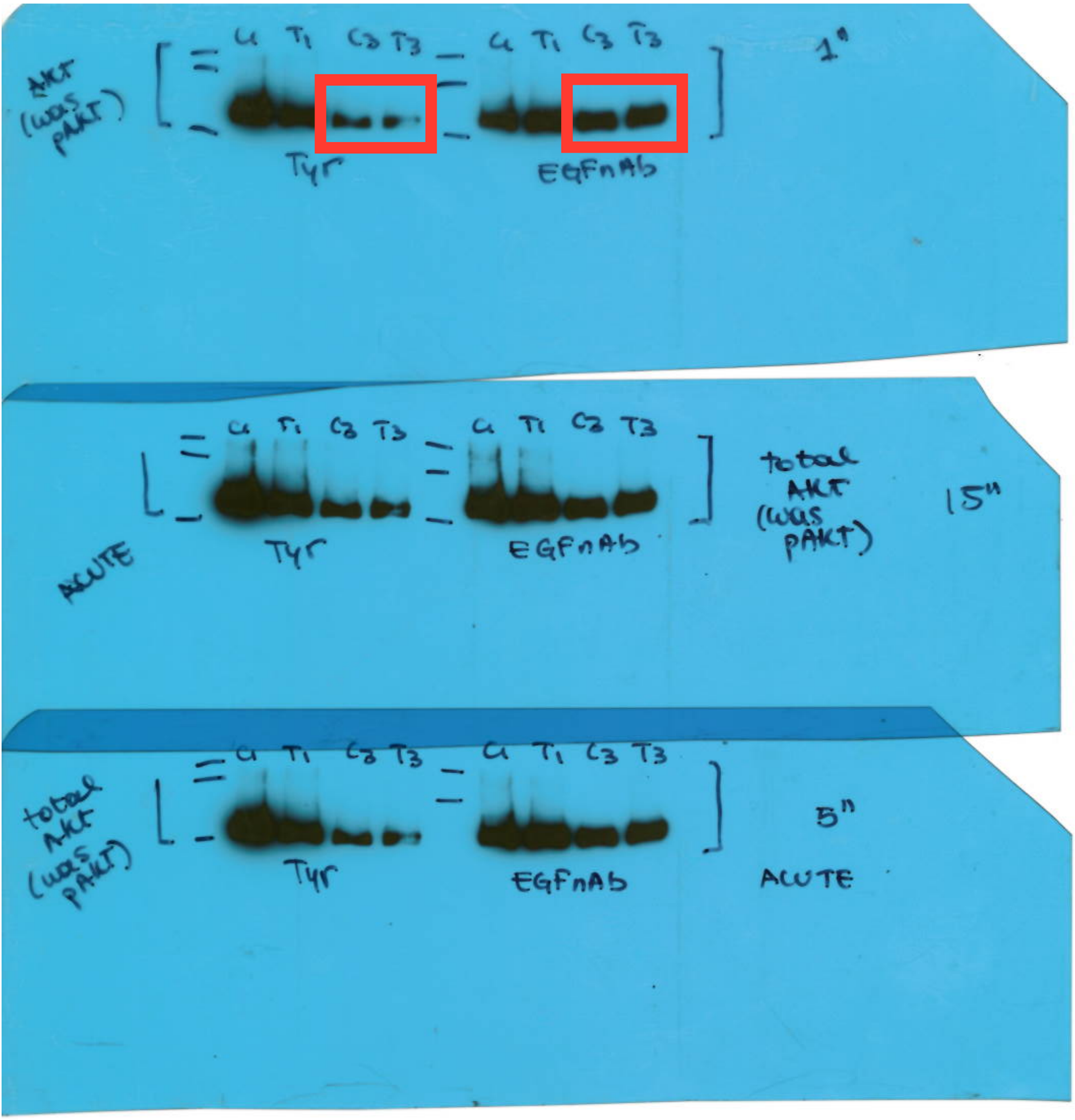

AKT (was pAKT)
C1 T1 C3 T3 C1 T1 C3 T3
1"
Tyr
EGFnAb
C1 T1 C3 T3 C1 T1 C3 T3
total AKT (was pAKT)
15"
Tyr
EGFnAb
ACUTE
total AKT (was pAKT)
C1 T1 C3 T3 C1 T1 C3 T3
5"
Tyr
EGFnAb
ACUTE

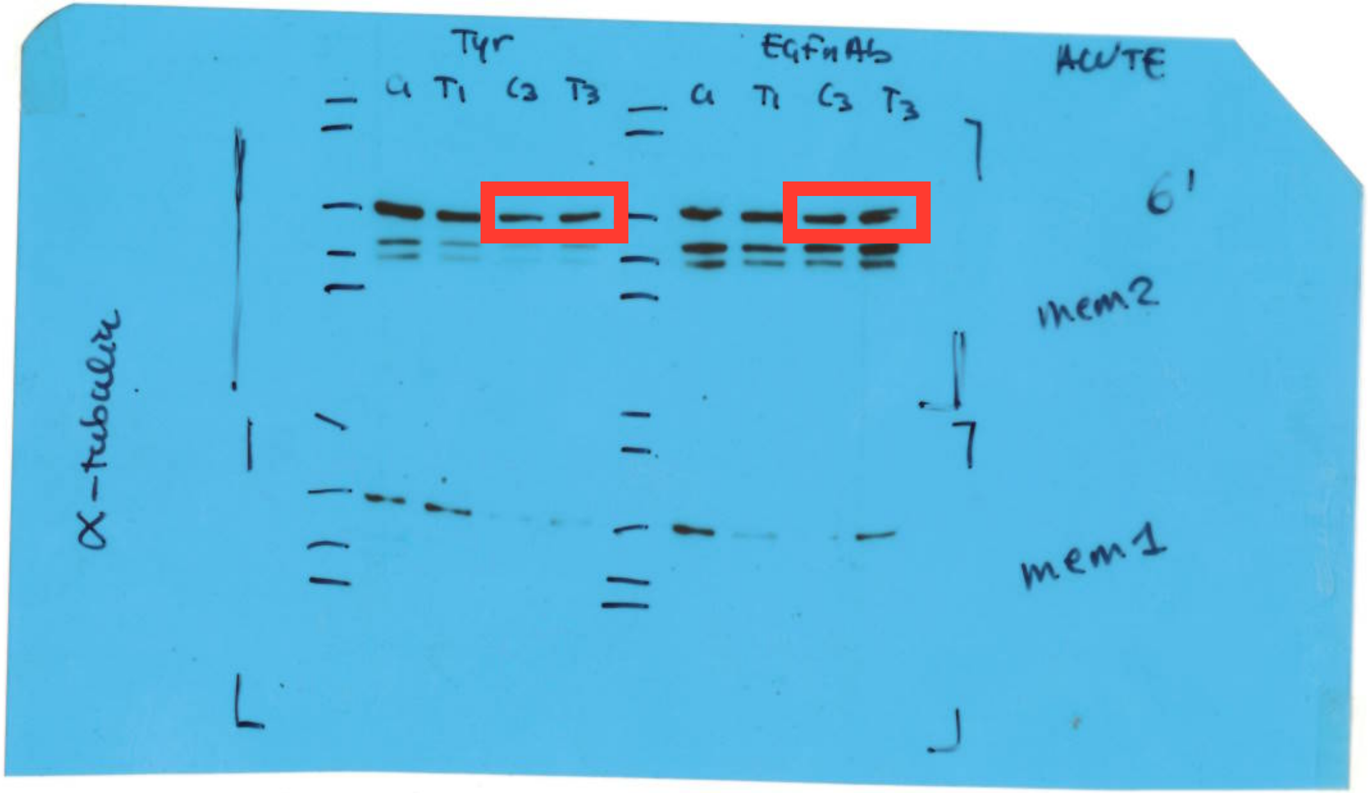
Tyr
C1 T1 C3 T3
EGFnAb
C1 T1 C3 T3
ACUTE
6'
mem2
mem1
α-tubulin

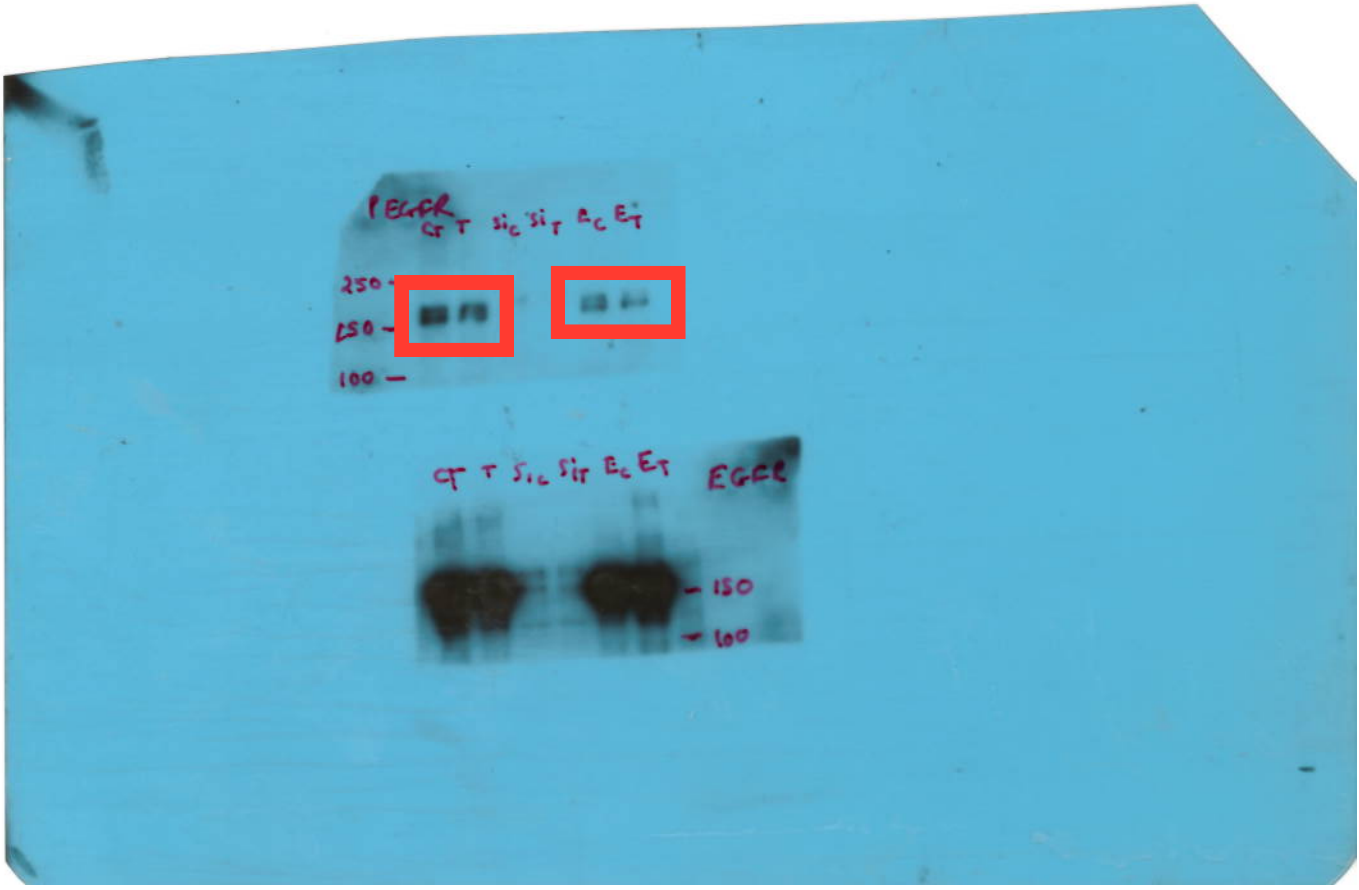
pEGFR
250
150
100
EGFR
150
100

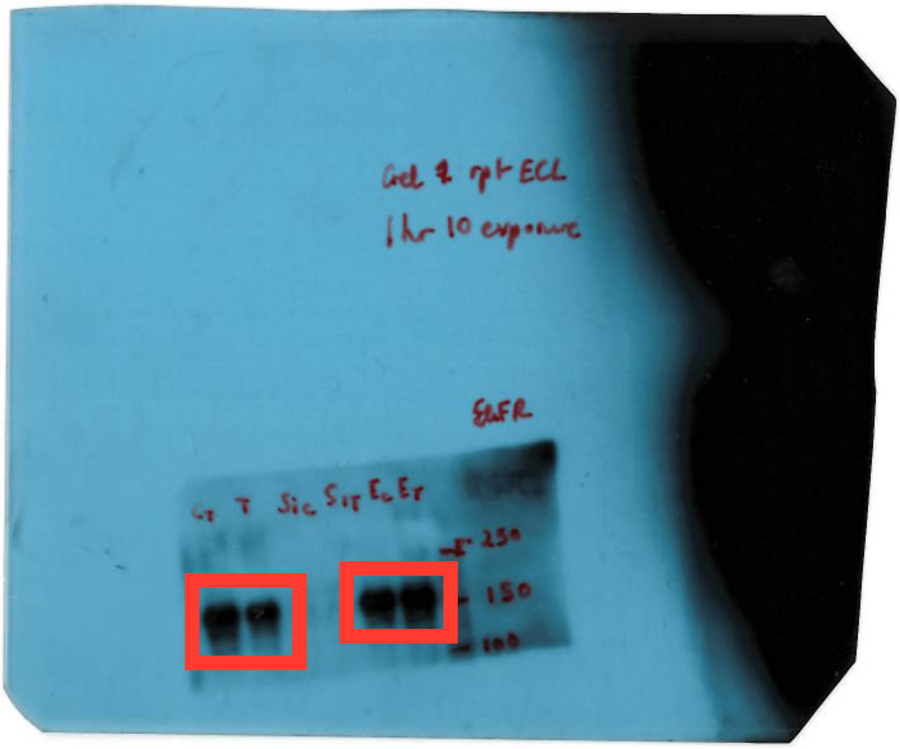
ECL
1 hr 10 exposure
T
250
150
100

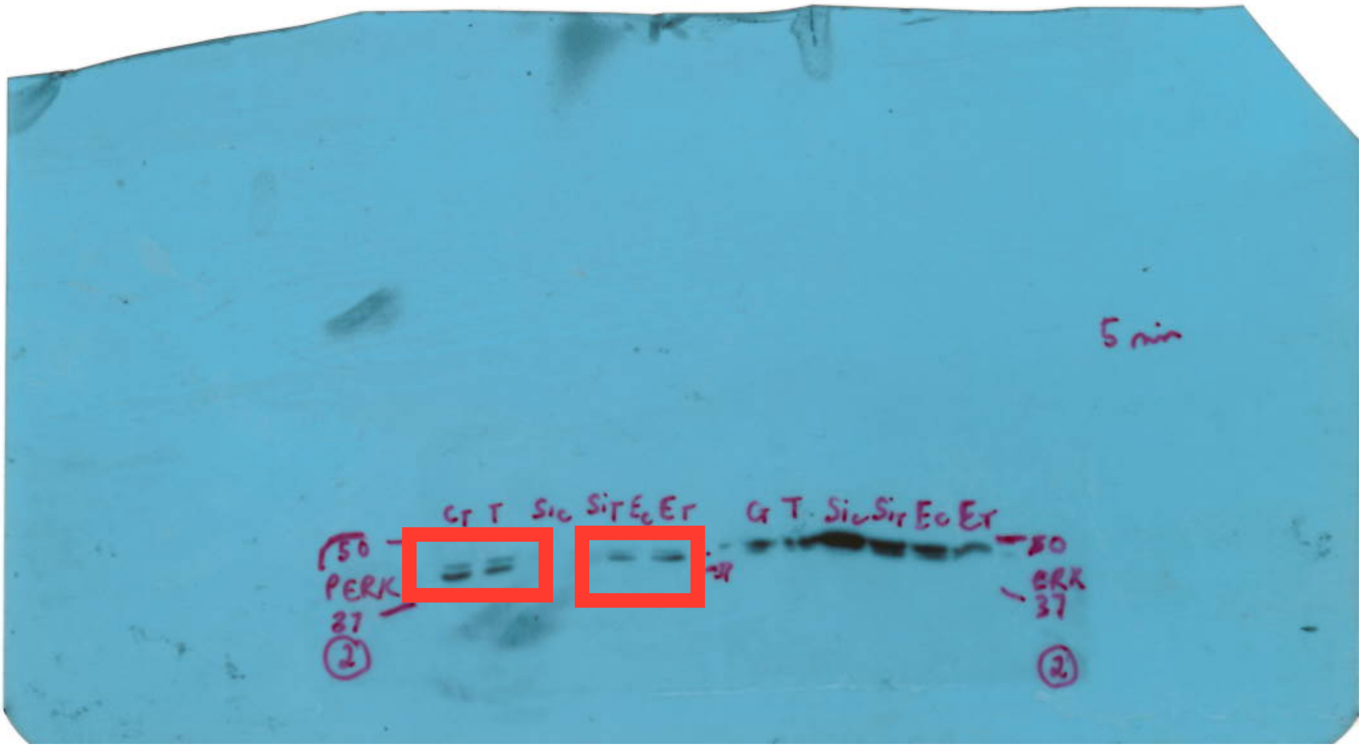
5 min
G T Sic SiT Ec Er
G T Sic Sir Ec Er
50
PERK
37
2
50
ERK
37
2

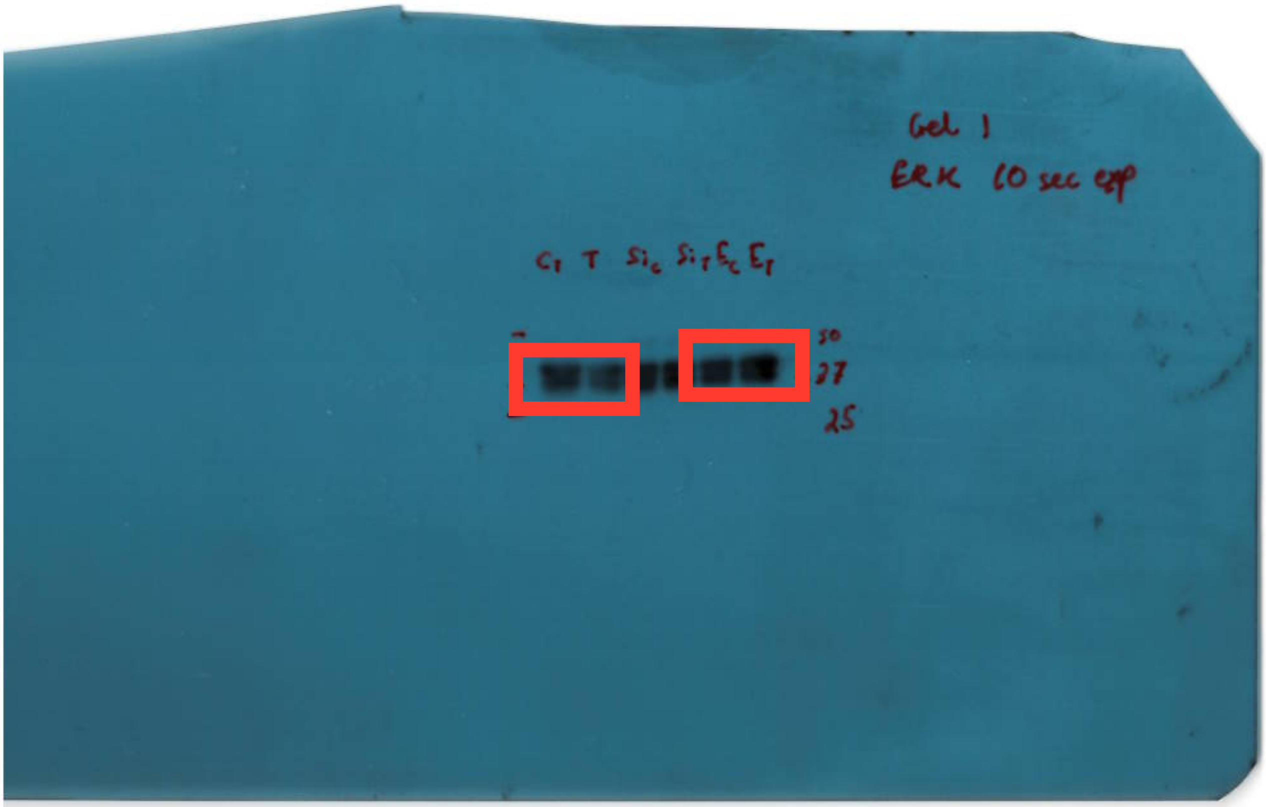
Gel 1
ERK 60 sec exp
C T Si Si E E
50
37
25

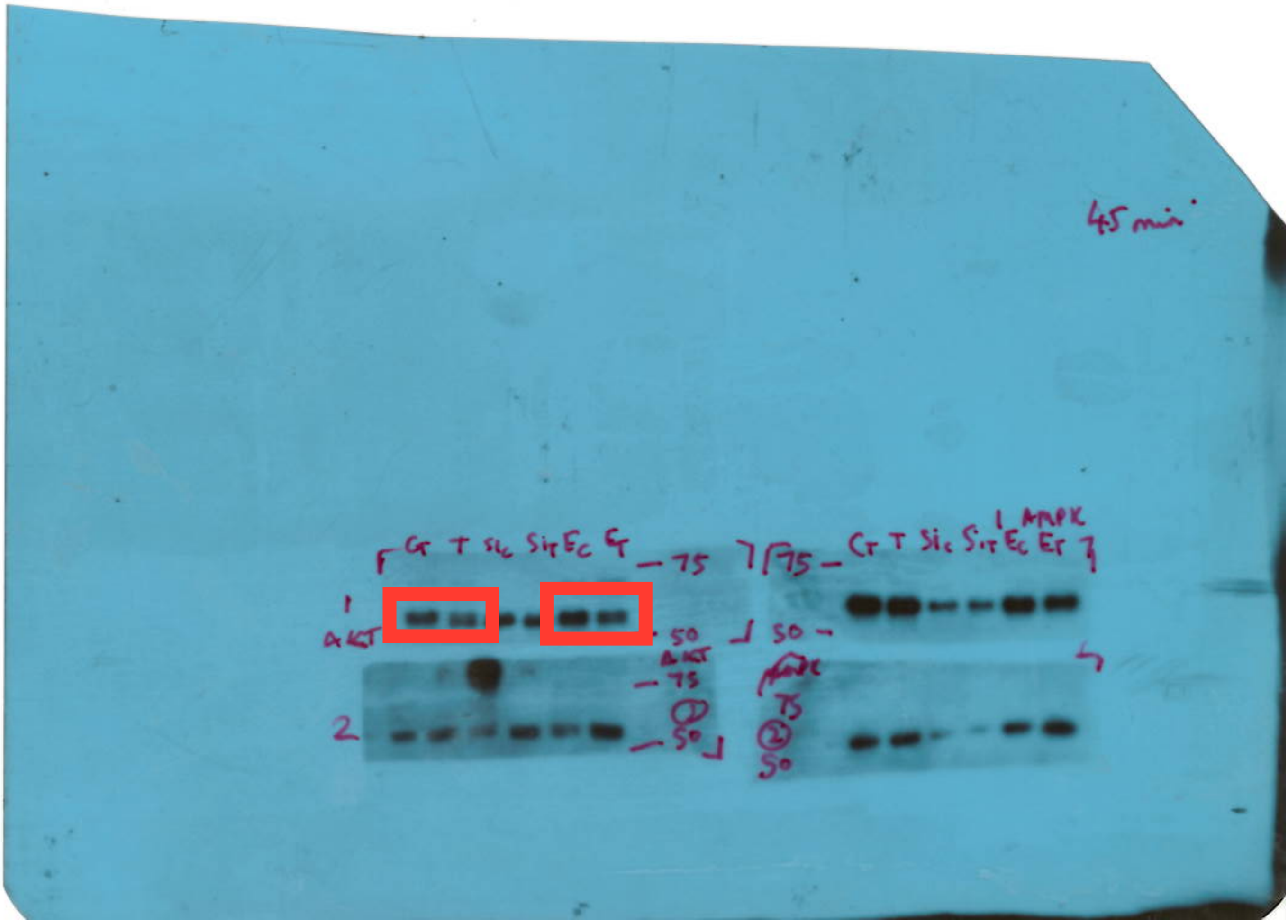

45 min
AKT
AMPK
75
50

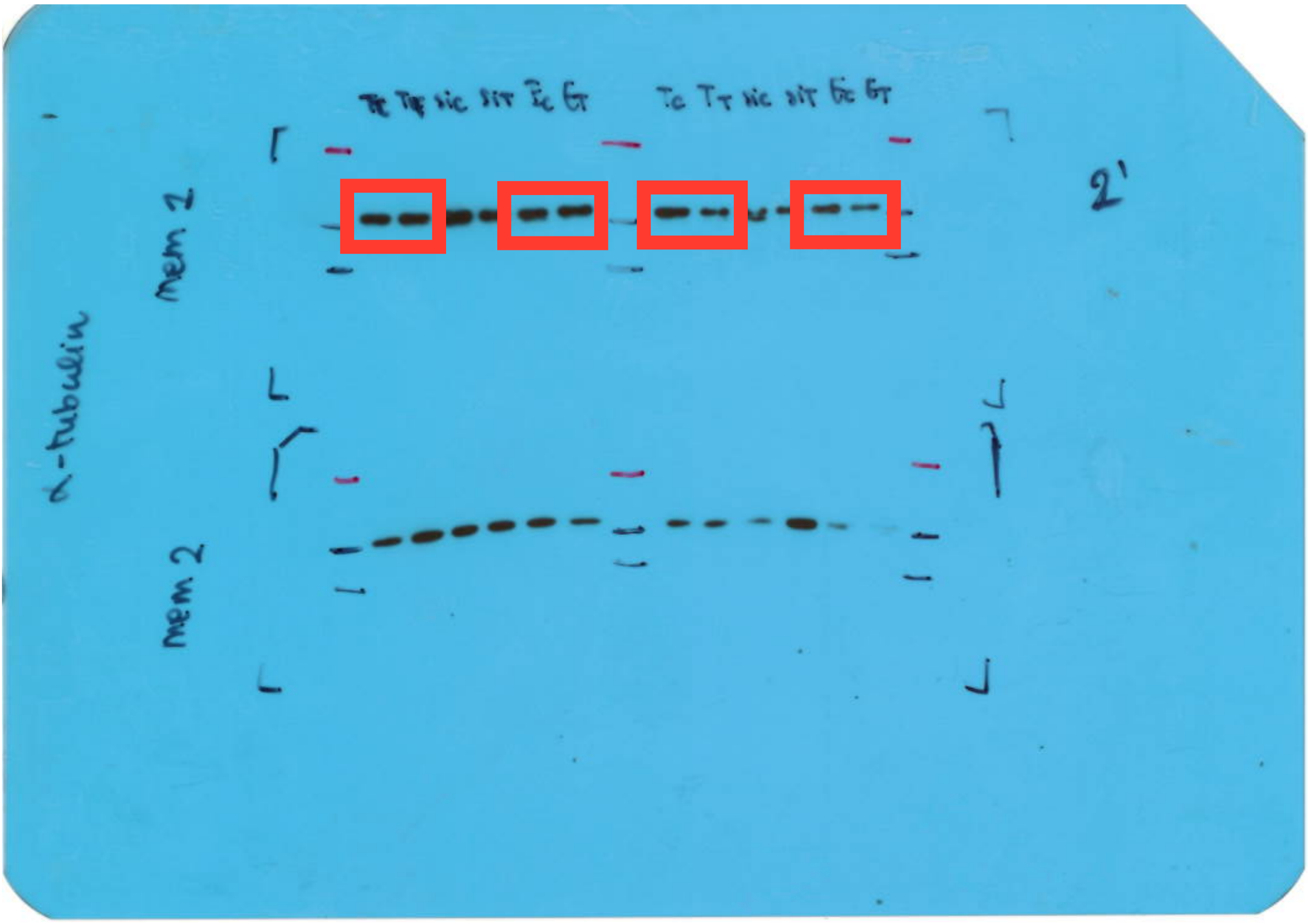
mem 1
mem 2
2'
