# Supplementary material for: EGF receptor (EGFR) inhibition promotes a slow-twitch oxidative, over a fast-twitch, muscle phenotype
Source: Sci Rep. 2019 Jun 25;9:9218. doi: 10.1038/s41598-019-45567-4 (PMC6592914; doi:10.1038/s41598-019-45567-4)
Supplement: Supplementary file 5 — Supplementary information excluding figures [file 41598_2019_45567_MOESM5_ESM.pdf]

SUPPLEMENTARY INFORMATION EXCLUDING FIGURES

LEGENDS FOR SUPPLEMENTARY FIGURES

Figure E1 Network driven by EGF signaling associated with repression of ST muscle phenotype identified by Ingenuity Pathway Analysis (IPA, Qiagen) of published mouse data

Network identified through the use of IPA (QIAGEN Inc., https://www.qiagenbioinformatics.com/products/ingenuitypathway-analysis(1)) Abbreviations: EGF epidermal growth factor, AMPK adenosine monophosphate kinase, TGFβ transforming growth factor beta, NFAT nuclear factor of activated T cells, nNOS neuronal nitric oxide synthase, FAK focal adhesion kinase, UVA-MAPK ultraviolet A mitogen activated protein kinase

Figure E2 Microarray data from COPD patient and control muscle identifying the module contribution to ST fibre proportion (A) and exercise capacity, and the relationship between expression of EGF (within the yellow module) and exercise capacity

Abbreviations: EGF epidermal growth factor, M module

Modeling demonstrated that the yellow and turquoise modules of transcript clusters contributed equally to ST fiber proportion (A) but that the yellow module made the largest contribution to exercise capacity, as measured by peak oxygen consumption on a maximal incremental cycle ergometry test (B). Quadriceps EGF transcript levels were correlated with exercise capacity in COPD patents alone, controls alone, and when both groups were combined (C).

Figure E3 Data on *EGF* and *EGFR* transcripts in quadriceps muscle and quadriceps endurance from COPD patients in small PCR cohort and serum EGF protein concentrations in large patient and control cohort

A-C qPCR data, normalized to B2 microglobulin transcripts, then converted to log2 scale from quadriceps biopsies and quadriceps endurance data (time to quadriceps fatigue (T_{80}) on standardized fatigue protocol (2, 3)) from small patient cohort D Serum protein EGF concentrations (quantified by ELISA) in 92 COPD patients and 16 healthy controls.

A: EGFR transcripts in COPD low and normal ST groups (median and interquartile range shown, Mann-Whitney U test used, p value not significant), B: EGF and EGFR transcripts (Pearson's r=0.29, p=0.28, ns), C: EGF transcripts and (Pearson's r=-0.64, p=0.01), and D: Serum EGF protein levels in COPD patients (split by having a low or normal ST proportion, and together) and controls (no significant difference between any groups).

Figure E4 Representative images of immunohistochemical staining for MyHC I protein in C2C12 mouse myotubes treated with an EGFR inhibitor, AG-1478, or vehicle control

C2C12 myotubes treated with AG-1478 or vehicle control were immunostained to assess expression of MyHC I protein (shown with green fluorescence, see supplemental methods) with nuclei stained with DAPI (see in blue). Staining of MyHC I protein (normalised for number of nuclei) was increased in cells treated with AG-1478 compared to vehicle control. Scale bar=50um

Figure E5 Representative images of immunohistochemical staining for MyHC I protein in C2C12 mouse myotubes derived from differentiating myoblasts transfected with an siRNA to the EGFR or scrambled vehicle control

See supplemental methods for details. MyHC I (green) and nuclei (DAPI, blue). Staining of MyHC I protein (normalised for number of nuclei) was increased in siRNA-transfected cells compared to negative control-transfected cells (nuclei stained with DAPI, seen in blue). Scale bar=50um

Figure E6 Western blot image of proteins extracted from C2C12 myotubes (n=2) and quantification of protein bands (n=3) 1 hour after AG1478 or EGF neutralizing antibody treatment or 3 days after transfection of EGFR siRNA or respective vehicle control (Fig A),1 hour after AG1478 or EGF neutralizing antibody treatment or respective vehicle control plus 100ng/ml recombinant EGF added to all conditions (Fig B), 24 hours after final treatment with AG1478, EGF neutralizing antibody or respective vehicle control (Fig C) and graph of quantification of bands in C (Fig D)

In both (A) and (B) phosphorylated (p) and total protein bands originate from same membrane but not in (C) where each phospho- and total protein band was quantified separately with

respective to alpha-tubulin to identify changes in both expression of total protein and phosphorylation at 3 day time-point. Quantifications were then expressed as fold change relative to control.

Figure E7 Representative images of MitoTracker® Red staining of mitochondria in C2C12 mouse myotubes treated with an EGFR inhibitor, AG-1478, or vehicle control
See supplemental methods for details. MitoTracker® Red staining of functional mitochondria (normalised for number of nuclei) was increased in cells treated with AG-1478 compared to vehicle control (nuclei stained with DAPI, seen in blue). Scale bar=50um

Figure E8 Representative images of MitoTracker® Red staining of mitochondria in C2C12 mouse cells transfected with an siRNA to the EGFR or scrambled vehicle control

MitoTracker® Red staining (normalised for number of nuclei) was increased in siRNA-transfected cells compared to negative control-transfected cells (nuclei stained with DAPI, seen in blue). Scale bar=50um

Figure E9 Images of in situ mRNA hybridization for *egfra* transcripts in 2 dpf zebrafish larva

Scale bar=100 μm

Figure E10 Schematic of initial nucleotide sequence of Exon 1 of the *egfra* gene in *Danio rerio* showing the region targeted by the single guide RNA

Nucleotides in italics indicate 5' UTR, bold uppercase: exon1, lowercase: initial part of intron 1.

Figure E11 Schematic of mutation and frameshift in exon 1 of *egfra* and truncated peptide sequence in the *egfra*$^{+/kg134}$ zebrafish with reference to the normal EGFR protein structure

Video E11: 3D reconstruction of WT larva at 2dpf showing immunostained ST fibers in somites 16-18

Fish shown in conventional orientation with head to the left, tail to the right, left side of the fish anteriorly and dorsum uppermost. WT siblings of *egfra*$^{+/kg134}$ larvae were were grown to 2 dpf, fixed and stained for slow MyHC (see supplementary methods). Z-stacks were acquired on 3 channels (633nm for ST fiber, and 461nm for nuclei) using a Zeiss LSM5 Exciter confocal microscope using 40X/NA1.1 immersion objective. Scale bar=50um

Video E12: 3D reconstruction of *egfra*$^{+/kg134}$ larva at 2dpf showing immunostained ST fibers in somites 16-18

Fish shown in conventional orientation with head to the left, tail to the right, left side of the fish anteriorly and dorsum uppermost. F2 *egfra*$^{+/kg134}$ males were crossed to tagged ß-actin: GFP (Tg(Ola.Actb:Hsa.HRAS-EGFP)^{vu119}) (4) females and embryos with GFP were grown to 2 dpf, fixed and stained for slow MyHC (see supplementary methods). Z-stacks were acquired on 3 channels (633nm for ST fiber, and 461nm for nuclei) using a Zeiss LSM5 Exciter confocal microscope using 40X/NA1.1 immersion objective. Scale bar=50um

SUPPLEMENTARY METHODS

PATHWAY ANALYSIS OF MOUSE DATA

Differentially expressed genes in isolated mouse ST and FT IIb fibers listed in the repository from Chemello *et al* (5) were entered into Ingenuity Pathway Analysis software (IPA, Qiagen). The significance of associations between the dataset and canonical pathways was calculated from the ratio of gene number from the mouse dataset mapping to the pathway to total gene number in that pathway, using a threshold of 0.1 and a p value of <0.05 as statistically significant.

PATIENT STUDY

The convenience sample of participants used for the microarray and validation study, and the methods used for clinical phenotyping, biopsy the quadriceps muscle, and analysis of fiber type proportion and cross-sectional areas are described in (2). Study ethics numbers are 06/Q0404/35 and 06/Q0410/54, approved by the Royal Brompton and Harefield NHS Foundation Trust and Ealing and West London Mental Health Trust Research Ethics

Committees respectively.

Microarray analysis

These are as described in (6) in press (doi: 10.1038/s41598-018-29789-6)

Gene expression profiling

Total RNA was extracted from 101 COPD and 21 healthy control quadriceps biopsies using the RNeasy fibrous tissue kit (Qiagen). RNA quality was determined by Bioanalyzer (Agilent), and samples with a RIN (RNA Integrity Number) <6 were excluded. RNA was prepared for whole transcriptome microarray analysis (98 COPD and 19 healthy controls) using the WT expression kit (Ambion) and GeneChip WT terminal labelling and hybridisation kit (Affymetrix). Samples were processed using the GeneTitan system and HuGene 1.1 ST 16- or 24-PEG array plates (Affymetrix).

Data analysis

Raw data quality was assessed using arrayQualityMetrics (3.22.1). Data were RMA-treated, summarizing at the level of Transcript Clusters (TC) using Affymetrix Power Tools (APT, version 1.16.1). Technical batch effects were identified via Principal Variance Component Analysis (PVCA 1.6.0), and the data adjusted accordingly using ComBat (SVA 3.12.0)(7). TC not forming part of the main array design, and universally lowly expressed TC were removed. Remaining outlying samples were specified through hierarchical clustering and removed, yielding 95 samples (79 COPD, 16 healthy controls) for analysis. Differential expression between healthy and COPD samples was estimated using *Limma* (3.22.7). P-values were adjusted for multiple testing using the Benjamini & Hochberg method (8) to control the False Discovery Rate (FDR) below 5% for Disease Ontology and Disease-Gene Associations (9). Occult patterns of transcriptional co-ordination were sought amongst differentially expressed genes through Weighted Gene Co-expression Network Analysis (WGCNA, 1.46)(15), employing a soft-thresholding power of 6 and minimum module size of 30. The relationship between modules and measured traits was assessed through robust biweight midcorrelation, with the exact number of observations taken into account in p-value calculation. Enrichment analysis was performed on unique entrez IDs using the Bioconductor packages topGO (2.20.0) for Gene Ontology and DOSE (3.0.4) for Disease Ontology and Disease-Gene Associations (16). Annotations were made using packages annotate (1.46.1) and hugene11sttranscriptcluster.db (8.3.1).

<u>RNA extraction, cDNA synthesis and qPCR</u> on smaller patient cohort (see below)

<u>Quantification of serum EGF levels by enzyme linked immunosorbent assay (ELISA)</u> Frozen

serum from patients and controls were thawed, diluted 1:10 and EGF protein concentrations (according to optimisation) and measured in quantified in pg/ml using a commercial kit (Quantikine ELISA, R&D systems) according to the manufacturer's instructions.

CELL CULTURE EXPERIMENTS

C2C12 mouse myotube culture

AG-1478 and EGF neutralizing antibody antibody (Santa Cruz, rabbit polyclonal anti-mouse EGF (# 06-102) experiments

The C2C12 mouse myoblast cell line, originally obtained through serial passage of myoblasts cultured from the thigh muscle of C3H mice after a crush injury (10) were grown and seeded as described previously (11). The following day standard (normal) growth medium (NM) was switched to mouse cell-differentiation medium (DM, DMEM supplemented with 10% (v/v) horse serum) to stimulate differentiation. DM was replaced every two days with DM. At day 8, cells were multinucleate, branching myotubes and were treated with drug or vehicle control daily for three days (days 8, 9 and 10). On Day 11 cells were lysed for RNA and protein extraction, or stained for MyHC I protein by immunofluorescence or for functional mitochondria with MitoTracker® Red (Invitrogen).

siRNA to EGFR experiment

As transfection of myotubes can be difficult (high risk of myotube contraction and peeling off plate and variable transfection efficiency), we seeded C2C12 myoblasts, and on day 2 when reaching 50% confluence, these were transfected with 1.5pmol/ul siRNA to EGFR (Ambion, Cat no. 4390771) or scrambled negative control (Ambion, negative silencer select, Cat no. 4390846) in serum-free NM as previously described (12) for 24 hours. After transfection, serum-free NM was replaced by DM and myoblasts differentiated into myotubes for three days (replacing DM on second day). There was no apparent difference in degree of differentiation from morphology of myotubes transfected with siRNA to EGFR or scrambled negative control. On Day 4, cells were lysed for RNA and protein extraction, or stained for MyHC I protein by immunofluorescence or for functional mitochondria with MitoTracker® Red (Invitrogen).

RNA extraction, cDNA synthesis and qPCR from cells and human muscle

Total RNA was isolated from C2C12 cells with TRIzol according to the manufacturer's instructions. The concentration of RNA was quantified using a spectrophotometer (Nanodrop ND1000, Wilmington, USA). First strand cDNA was generated using Ominiscript RT No 1010890 (Qiagen, UK). Real-time quantitative PCR (RT-qPCR) analysis was carried out in duplicate on each cDNA sample for both the target gene and for the reference housekeeping

gene. A 10 µl reaction of FAST Sybr (Qiagen, UK) and the primer pair (2pmol/µl) in 96 well plates (MicroAmp, Fast optical 96 well reaction plate (0.1 ml) (Applied Biosystems, UK) was run. QPCR reactions were run on the 7500 Fast Real-time PCR System (Applied Biosystems, UK.), with the following cycle program: 95 °C for 10 minutes, then 40 cycles of 95 °C for 10 seconds, 60°C for 30 seconds. Melt curve analyses were run to ensure a single product; also confirmed by running PCR products on a 2% agarose gel to confirm a single product of the expected size. Cycle threshold values for target genes and housekeeping genes were utilised to obtain normalised values of target genes (arbitrary units, AU). Housekeeping genes were selected on the basis that there was no systematic and statistically significant difference in the cycle threshold values of the gene between control and treated cells. HPRT was used as the housekeeper for all cell experiments except the PD98059 experiment where HPRT was systemically affected by treatment therefore 5S was used. Beta 2 microglobulin was used as the housekeeper for the human PCR data.

Table 1 Mouse, human and zebrafish primers used for quantitative RT-PCR analysis

Gene	Forward primer	Reverse primer
Mouse		
MYH7	5’-TGTGATAGCCTTCTTGGCCT-3’	5’-AGCAGGAGCTGATTGAGACC-3’
MYH2	5’-CAGCTTGTTGACCTGGGACT-3’	5’-TTGGTGGATAAACTCCAGGC-3’
MYH1	5’-AGCTTGTTGACCTGGGACTC-3’	5’-ACCTTGTGGACAAACTGCAA-3’
MYH4	5’-GTTTGTCCACCAAGTCCTGC-3’	5’-ACCTTGTGGACAAACTGCAA-3’
UCP3	5’-GGAGTCTCACCTGTTTACTGACAACT-3’	5’-GCACAGAAGCCAGCTCCAA-3’
CS	5’-GGAAGGCTAAGAACCCTTGG-3’	5’-TCATCTCCGTCATGCCATAGT3’
GLUT4	5’-CCGCGGCCTCCTATGAGATAC-3’	5’-GATGAGTGGGGGCGATTTCTC-3’
HPRT	5’-GCAGTACAGCCCCAAAATGG-3’	5’-AACAAAGTCTGGCCTGTATCCAA-3’
B2M	5’-CCGTCTACTGGGATCGAGAC-3’	5’-GCTATTTCTTTCTGCGTGCAT-3’
5S-rRNA	5’-ATCTCGGAAGCTAAGCA-3’	5’-GGTCTCCCATCCAAGTACT-3’
Human		
EGF	5’-CCTTATGAGGAGTCGAGCCAGA-3’	5’-CCTGCCTCCATGAAGTTGGTT-3’
EGFR	5’-GGTGACTCCTTCACACATAC-3’	5’-TTGGTCCTGCCGCGTATGAT-3’
B2M	5’-TGCTGTCTCCATGTTTGATGTATCT-3’	5’-TCTCTGCTCCCCACCTCTAAGT-3’
Zebrafish		

EGFR	5'-ACGAGACCACTGAACGCTTA-3'	5'-TGCTCCCTGACACACTTTCT-3'
SOX6	5'-GCGCATGGAATCGGACAG-3'	5'-GGCTTGTGTGGAGAGGTAGAG-3'
smyhc1	5'-CATGCAGAAAGACTAAGGGA-3'	5'-TGGTGTTATGCTAAGGACAC-3'
mylz2	5'-TCCCTTTCTTGCTTTCTACC-3'	5'-GAAAACAATCCAATGTCCCC-3'
B2M	5'-TTGGCTCTCTCGAATAAAAC-3'	5'-CTTTCGGAGTGGAGACTTTC-3'
TBP	5'-GATCCTGCGAATTATCGTTT-3'	5'-TTCAGGGCTTCCCTATAAAA-3'
Mt-nd1	5'-AGCCTACGCCGTACCAGTATT-3'	5'-GTTTCACGCCATCAGCTACTG-3'
Polg1	5'-GAGAGCGTCTATAAGGAGTAC-3'	5'-GAGCTCATCAGAAACAGGACT-3'

Immunohistochemical staining for MyHC I protein in C2C12 myotubes

Cells grown on 96-well plate were fixed with 4% paraformaldehyde in PBS. Fixed cells were permeabilized with 0.3% Triton in 1xPBS for 15 min and blocked 5% bovine serum albumin. Cells were immunostained with BA-F8 anti-MHC I (1:50, DSHB Cat# BA-F8, RRID: AB_10572253), overnight at 4°C. Cells were incubated with anti mouse IgG1 Alexa 647 (1:500, IMGENEX Cat# 20109AF647, RRID: AB_1930712) at room temperature for 5h. Nuclei were stained with DAPI (1/10000). Pictures were acquired with Inverted Widefield Microscope with LED illumination Zeiss Axio Observer inverted microscope, stage was calibrated and multi-position acquisition mode was used to take 9 pictures per well. Single images were opened and analysed with imageJ, the integrated density value of the red channel was divided per the number of nuclei.

MitoTraker Red CMXRos staining of functional mitochondria (based on membrane potential)

Cells, grown on 96-well plate, were incubated with 50 nM of MitoTracker® Red CMXRos (Invitrogen) at 37°C for 20 min. Cells were fixed with 4% paraformaldehyde in PBS and stained with DAPI (1/10000). Images were acquired using a Zeiss Axio Observer Inverted microscope with LED illumination, the stage was calibrated and multi-position acquisition mode was used to take 9 pictures per well. Single images were opened and analysed with ImageJ, with the integrated signal from the red channel being divided by the number of nuclei present in the image.

Western blotting

Cells were lysed for 10 min in ice-cold cell lysis buffer (Cell Signaling Technology, Cat. No 9803) plus protease inhibitor cocktail (Sigma, Cat. No P8340) at 1:200 volume. Protein concentrations were quantified by Bradford assay. Protein extracts were mixed with 20 µl of 5× SDS-PAGE sample buffer with 2-mercaptoethanol and heated for 5 min at 100 °C, and 5-60 µg of protein (depending on the experiment and target for immunoblotting) were loaded on 4% stacking and 8% separating SDS-PAGE mini-gel. Electrophoresis was performed at

constant current (140V for 1.5-mm-thick gel). After electrophoresis, the proteins were electroblotted (wet transfer, 200mA for 2 hrs onto a 0.45-µm pore size PVDF membrane (Whatman, GE Healthcare). Subsequent steps were performed at room temperature, and antibody dilutions all in 5% milk in TBST (0.05% Tween). Transferred proteins were stained briefly with 0.1% Ponceau S (w/v) in 5% acetic acid (Sigma) to check for even loading and transfer. Membranes were blocked in 5% non-fat milk powder (w/v) in TBST (10 mM Tris/Cl, pH 7.4, 150 mM NaCl, and 0.05% Tween 20) for 1 h, washed three times for 15 min with TBST, treated for 1 h with primary antibody, then washed three times for 15 min with TBST, and incubated for 1 h with appropriate horseradish peroxidase-conjugated secondary IgG (Sigma). Immunodetection of proteins was visualized with a chemiluminescent detection reagent kit (Amersham ECL reagent cat no: RPN 2106). For protein extracted from cells lysed 1-hour post-single dose and from myotubes derived from cells transfected with siRNA to EGFR or scrambled control, phosphorylated proteins were probed first then membranes stripped of primary and secondary antibody using Antibody Striping Solution (Alpha Diagnostic Internation, Cat. No 90101) and then re-probed for total protein using the same technique. Therefore phospho-to-total EGFR, ERK and Akt ratios for the same loading sample was determined for control and treated specimens. Immunoblotting for alpha-tubulin was also performed as a loading control although for 1hour post-single dose total protein levels were unlikely to have occurred. Western blotting of phosphorylated and total protein levels extracted from cells on day following final treatment were probed on separate membranes and normalised to alpha tubulin as a loading control.

Protein quantities loaded per well

Sample	Proteins immunoblotted	Protein loaded [µg]
1 day post-final AG-1478 or vehicle control treatment	All	10
1 day post-final EGFnAb or vehicle control treatment	All	10
3 days following siRNA or scrambled control transfection	All	10
1 hr post-AG-1478 or vehicle control treatment	pEGFR, EGFR	60
1 hr post-AG-1478 or EGFnAb or vehicle control treatment	All except pEGFR, EGFR	15
1 hr post-AG-1478 or EGFnAb plus 10ng/ml EGF or vehicle control plus 10ng/ml EGF treatment	pEGFR, EGFR	60
1 hr post-AG-1478 or EGFnAb plus 10ng/ml EGF or vehicle control plus 10ng/ml EGF treatment	All except pEGFR, EGFR	15

EGFnAb: EGF neutralizing Ab

Primary Antibodies	Source (all USA)	Dilution
Anti-phospho-p44/42 MAPK (rabbit polyclonal)	Cell Signaling Technology (CST)	1:1000
Anti-p44/42 MAPK (rabbit polyclonal)	CST	1:1000
Anti-phospho-EGFR (rabbit monoclonal)	CST	1:1000
Anti-Akt (rabbit monoclonal)	CST	1:1000
Anti- phospho-Akt (rabbit polyclonal)	CST	1:500
Anti-EGFR (rabbit polyclonal)	Santa Cruz Biotechnology	1:500
Anti-alfa-tubulin (monoclonal mouse)	Sigma-Aldrich	1:10 000

Secondary Antibodies	Source	Dilution
Anti-rabbit (polyclonal Goat)	Deko, Denmark	1:3000
Anti-mouse (polyclonal sheep)	GE Healthcare, UK	1:10000

ZEBRAFISH STUDY

Zebrafish lines, maintenance and embryo manipulation

Zebrafish on the AB background were housed and manipulated under UK Home Office Licence and maintained at 26.5ºC under standard procedures (13). Embryos were microinjected at 1-2 cell stage and raised at 28.5ºC.

In situ hybridisation of *egfra* mRNA in zebrafish embryos

Whole-mount in situ hybridization with digoxigenin-labelled mRNA sense and antisense probes to *egfra* was performed as previously described (14). Briefly, zebrafish *egfra* cDNA from exon 1 to exon 7 was used as template to generate the probes, T3 or T7 sites were introduced at the 5'-end of the primers (forward 5'-TAATACGACTCACTATAGGGAGAAACGCTTACAAAGCCTGGAA-3' and reverse 5'-GGATCCATTAACCCTCACTAAAGGGAACGTGATCCGTCACCACATAG-3') allowing direct *in vitro* transcription of purified PCR- fragments. The PCR product was transcribed *in vitro* using T7 and T3 polymerase to generate sense and anti-sense probes, respectively, which were purified with Illustra ProbeQuant G-50 Micro Columns (GE Healthcare, Code: 28-9034-08) according to the manufacturer's instruction. Two dpf embryos were hybridized overnight at 65°C with 1 ng/uL of sense or antisense probe (dilution 1:100). Embryos were mounted in glycerol and photographed as a wholemounts with a Leica DFC490 camera.

Mutation of *egfra* using CRISPR/Cas9 system

Single guide RNA (sgRNA) was designed using crispr direct (https://crispr.dbcls.jp) which scans targets areas for 20 bp sequences plus a terminal NGG (PAM sequence), with prioritisation of a GA sequence at the 5' to ensure high efficacy of SP6 RNA polymerase for sgRNA synthesis. Oligonucleotides corresponding to the sgRNA (forward 5'-AGCTCCCGGAGAGCAATA-3' and reverse 5'-TATTGCTCTCCGGGAGCT-3'), plus the appropriate flanking nucleotides to allow ligation reaction with pDC039 vector, were annealed and then cloned into the linearized vector. To synthesis sgRNA, 1 µg of plasmid template was used for the SP6 *in vitro* transcription reaction (SP6 RiboMAX large scale RNA production kit, Promega, cat#P1280). Samples were DNase treated and the total RNA purification kit (Norgen, cat# 17200, 37500) protocol was adapted to purify and isolate the sgRNA. All steps were performed at room temperature. To bind the gRNA to the kit provided column, 100 µl of transcription reaction was mixed with 3 volumes of RL buffer and 2 volumes of 100% ethanol, mixed and transferred to the column, which was centrifuged at 6000rpm for 1 min and the flow-through was discarded. Column was washed 3 times by applying 400 µl of wash buffer A, centrifuged at 13000rpm for 1 min and discarding the flow-through. To elute the sgRNA, the column was placed in a clean elution tube and 30 µl of elution solution A applied, sgRNA

was collected by centrifugation at 14000rpm for 2 min. gRNA was quantified by QuBit (Invitrogen).

One-cell stage zebrafish embryos (AB background) were injected with a mixture containing 324 ng of Cas9-encoding mRNA, 120 ng of sgRNA and 5% phenol red. Successfully injected embryos were screened for mutation efficiency by High Resolution Melting analysis (HRMA) with primers designed to amplify the region across the target site (forward 5'-CCCGATAGCTTACAAACGCA-3' and reverse 5'-GCCGTTTCACAATAGTCCTACC-3'). Zebrafish founders and progeny were bred by outcrossing onto AB background. F3 generation heterozygote mutants and their WT siblings were used for phenotypic analysis.

DNA extraction and genotyping from 2 dpf zebrafish larvae

Mutation in the first generation of founders was identified by HRMA and confirmed by sequencing. DNA was extracted from tail finclips or the whole embryo incubated with lysis buffer (25 mM NaOH, 0.2 mM EDTA) for 45 min at 85°C following addition of an equal volume of neutralizing buffer (40 mM Tris-HCl pH 8). Three mutant lines were generated; the one selected for further analyses (allele number kg134) had a 1 base pair deletion, 2 base pair insertion, and 1 base pair substitution (see below). DNA extraction for mitochondrial DNA quantification was done from DNA extracted from whole larvae.

RNA extraction, cDNA synthesis and qPCR

Two dpf larvae were incubated in 1.5 ml tube with 100 ml lysis buffer containing 20 mg/ml of Proteinase K (ThermoFisher, #BP1700) for 10 min, 3 ml of the tube content was used for genotyping and the rest incorporated with 30 ml of TRIzol. Five 2 dpf fish with the same genotype were pooled and processed for RNA extraction with RNA Clean & Concentrator-5 kit (Zymo Research, #R1015) according to manufacturer's instructions. 150 ng of RNA were retro-transcribed with first strand cDNA generated using SuperScript III (Invitrogen, UK). Reverse transcriptase and qPCR protocol followed was identical to that used for cell experiments (see above). Target gene mRNA levels were normalised to geomean of B2-microglobulin and tata binding protein (TBP), which were stable between WT and heterozygote siblings. Mitochondrial DNA content was calculated by qPCR for *mt-nd1* (mitochondrial NADH dehydrogenase 1) normalised to nuclear DNA copies of *Polg* (DNA polymerase gamma) as previously described (15).

Immunostaining of ST fibers

F2 *egfra*$^{+/kg134}$ males were crossed to tagged ß-actin: GFP Tg(Ola.Actb:Hsa.HRAS-EGFP)^{vu119} line (4)) females and embryos with GFP-positive signal were grown for 2 days then fixed with 2% PFA for 30 min at room temperature (RT), washed and bleached with solution of 33% H_20_2, 59.5% H_2O, 5% formamide and 2.5% of 20x saline-sodium citrate buffer (SSC). Whole

fish were immunostained with A4.1025 (IgG2a, recognises all MyHC, used 1:10 (16); SHB Cat# A4.1025, RRID:AB_528356) overnight in primary antibody at 4°C in 2% goat serum, washed in PBS-Triton X-100 (0.1%) embryos were incubated overnight at 4°C with goat anti-mouse IgG2a Alexa633 (1:500, Invitrogen) and DAPI (1:1000, Sigma-Aldrich) in 2% goat serum. Antibody penetration into whole fish mounts allowed reliable detection of the outer ST fiber layer and counting of these fibers. After staining, the head of the fish was removed and DNA extracted genotyping. Tails were mounted laterally on glass slides in Citifluor (AF1) and imaged on a Zeiss LSM5 Exciter confocal microscope using 40X immersion objective. For each tail, Z-stacks were acquired in 2 channels (633nm for ST fiber and 405nm for nuclei).

To count the number of ST fibers a 3D projected of maximum intensity projection (Figure 3A) was obtained using Image J software (17). Fibers of wild type sibling (WT, n=16) and *egfra*$^{+/kg134}$ (n=16) were counted blind; once counting was completed, DNA was sent for genotyping by Eurofins genomic (www.eurofinsgenomics.eu).

REFERENCES

1. Kramer A, Green J, Pollard J, Jr., and Tugendreich S. Causal analysis approaches in Ingenuity Pathway Analysis. *Bioinformatics (Oxford, England).* 2014;30(4):523-30.
2. Natanek SA, Gosker HR, Slot IG, Marsh GS, Hopkinson NS, Man WD, et al. Heterogeneity of quadriceps muscle phenotype in chronic obstructive pulmonary disease (Copd); implications for stratified medicine? *Muscle & nerve.* 2013;48(4):488-97.
3. Swallow EB, Gosker HR, Ward KA, Moore AJ, Dayer MJ, Hopkinson NS, et al. A novel technique for nonvolitional assessment of quadriceps muscle endurance in humans. *Journal of applied physiology.* 2007;103(3):739-46.
4. Cooper MS, Szeto DP, Sommers-Herivel G, Topczewski J, Solnica-Krezel L, Kang HC, et al. Visualizing morphogenesis in transgenic zebrafish embryos using BODIPY TR methyl ester dye as a vital counterstain for GFP. *Developmental dynamics : an official publication of the American Association of Anatomists.* 2005;232(2):359-68.
5. Chemello F, Bean C, Cancellara P, Laveder P, Reggiani C, and Lanfranchi G. Microgenomic analysis in skeletal muscle: expression signatures of individual fast and slow myofibers. *PloS one.* 2011;6(2):e16807.
6. Saffron AG Willis-Owen AT, Paul Kemp, Michael I Polkey, William OCM Cookson, Miriam F Moffatt, Samantha A Natanek COPD is accompanied by co-ordinated transcriptional perturbation in the quadriceps affecting the mitochondria and extracellular matrix. *Scientific reports.* 2018:in press.

7. Johnson WE, Li C, and Rabinovic A. Adjusting batch effects in microarray expression data using empirical Bayes methods. *Biostatistics.* 2007;8(1):118-27.

8. Benjamini Y, and Hochberg Y. Controlling the false discovery rate: a practical and powerful approach to multiple testing. *Journal of the Royal Statistical Society Series B.* 1995(57):289-300.

9. Yu G, Wang L-G, Yan G-R, and He Q-Y. DOSE: an R/Bioconductor package for disease ontology semantic and enrichment analysis. *Bioinformatics (Oxford, England).* 2015;31(4):608-9.

10. Yaffe D, and Saxel O. Serial passaging and differentiation of myogenic cells isolated from dystrophic mouse muscle. *Nature.* 1977;270(5639):725-7.

11. Martin KM, Cooper WN, Metcalfe JC, and Kemp PR. Mouse BTEB3, a new member of the basic transcription element binding protein (BTEB) family, activates expression from GC-rich minimal promoter regions. *The Biochemical journal.* 2000;345 Pt 3:529-33.

12. Favot L, Hall SM, Haworth SG, and Kemp PR. Cytoplasmic YY1 is associated with increased smooth muscle-specific gene expression: implications for neonatal pulmonary hypertension. *Am J Pathol.* 2005;167(6):1497-509.

13. Westerfield M. *The Zebrafish Book. A Guide for the Laboratory Use of Zebrafish (Danio rerio).* University of Oregon Press, Eugene; 2000.

14. Hinits Y, and Hughes SM. Mef2s are required for thick filament formation in nascent muscle fibres. *Development (Cambridge, England).* 2007;134(13):2511-9.

15. Artuso L, Romano A, Verri T, Domenichini A, Argenton F, Santorelli FM, et al. Mitochondrial DNA metabolism in early development of zebrafish (Danio rerio). *Biochimica et biophysica acta.* 2012;1817(7):1002-11.

16. Hinits Y, Williams VC, Sweetman D, Donn TM, Ma TP, Moens CB, et al. Defective cranial skeletal development, larval lethality and haploinsufficiency in Myod mutant zebrafish. *Developmental biology.* 2011;358(1):102-12.

17. Schindelin J, Arganda-Carreras I, Frise E, Kaynig V, Longair M, Pietzsch T, et al. Fiji: an open-source platform for biological-image analysis. *Nature methods.* 2012;9(7):676-82.
